# The Association between Maternal Oral Inflammation and Neutrophil Phenotypes and Poly-Unsaturated Fatty Acids Composition in Human Milk: A Prospective Cohort Study

**DOI:** 10.3390/cells11244110

**Published:** 2022-12-17

**Authors:** Rana Badewy, Amir Azarpazhooh, Howard Tenenbaum, Kristin L. Connor, Jim Yuan Lai, Michael Sgro, Richard P. Bazinet, Noah Fine, Erin Watson, Chunxiang Sun, Sourav Saha, Michael Glogauer

**Affiliations:** 1 Faculty of Dentistry, University of Toronto, Toronto, ON M5G 1X3, Canada; 2 Department of Dentistry, Centre for Advanced Dental Research and Care, Mount Sinai Hospital, Toronto, ON M5G 1X5, Canada; 3 Department of Health Sciences, Carleton University, Ottawa, ON K1S 5B6, Canada; 4 Department of Pediatrics, St. Michael’s Hospital, Unity Health Toronto, Toronto, ON M5B 1W8, Canada; 5 Department of Pediatrics, University of Toronto, Toronto, ON M5G 1X3, Canada; 6 Department of Nutritional Sciences, University of Toronto, Toronto, ON M5S 1A8, Canada; 7 Matrix Dynamics Group, Faculty of Dentistry, University of Toronto, Toronto, ON M5G 1X3, Canada; 8 Department of Dental Oncology, Princess Margaret Cancer Centre, Toronto, ON M5G 2C1, Canada

**Keywords:** neutrophils, breast milk, oral inflammatory load, periodontal diseases, oral polymorphonuclear neutrophils, human milk, breastfeeding, periodontitis, gingivitis

## Abstract

This prospective cohort study aimed to investigate the impact of maternal oral inflammation on human milk composition including neutrophil counts, activation state (based on cluster of differentiation (CD) markers expression), and fatty acid levels. Fifty mothers were recruited from St. Michael’s hospital, Toronto, and followed up from 2–4 weeks until 4 months postpartum. Oral rinse and human milk samples were collected at both timepoints. Oral polymorphonuclear neutrophils (oPMNs) within the rinses were quantified using flow cytometry and the participants’ oral health state was categorized into three groups (i.e., healthy, moderate, and severe) based on the oPMNs counts. Fatty acids were identified and quantified using a gas chromatography-flame ionization detector (GC-FID). Compared to mothers with a healthy oral health state, mothers with moderate to severe oral inflammation had a statistically significant decrease in the expression of CD64 biomarker, an increase in the expression of CD14 biomarker on human milk neutrophils and a decrease in the levels of eicosapentaenoic acid (C20:5n-3) in their human milk at follow-up compared to baseline. This study demonstrates for the first time that maternal oral inflammation can affect human milk composition. The mechanism by which these alterations can affect infant health outcomes in the long term critically needs to be considered.

## 1. Introduction

Inflammatory oral health conditions, including periodontal diseases, can affect women during the pregnancy and postpartum periods [1,2,3]. Throughout the postpartum period, women tend to be affected by a continuous deterioration of their periodontal health if pre-existing oral health problems remain untreated [2,4]. In relation to oral inflammatory diseases, there has been a groundswell of data showing that oral and systemic health conditions can be correlated to one another, including the negative impact of poor oral health on pregnancy outcomes (e.g., low birth weight, premature labour) [1,5], cardiovascular diseases [6], and diabetes [7]. However, it is still unclear as to whether maternal oral diseases can affect the immunological and nutritional components of human milk including its neutrophil counts, their states of activation, as well as the content of fatty acids (FAs or fatty acid; FA).

Periodontal diseases are inflammatory in nature [8] and recently, novel approaches have been developed and validated for early detection of oral inflammation levels that correlate with the severity of periodontal disease by using well-characterized oral rinses that detect oral polymorphonuclear neutrophil (oPMNs) levels [8,9,10,11]. Quantification of oPMNs as markers of periodontal inflammation [a concept known as oral inflammatory load (OIL)] [8,10,11] has been shown recently to correlate well to the degree and extent of clinical markers for periodontitis amongst pregnant women [9].

Polymorphonuclear leukocytes (PMNs) are the most common white blood cells, accounting for 50–70% of all circulating leukocytes [8,10]. Several critical functions make PMNs the first line of defence against bacterial pathogens, including the phagocytosis of invading pathogens, degranulation and the release of antimicrobial peptides, release of reactive oxygen species and the formation of neutrophil extracellular traps [12,13,14,15]. It has also been shown that oPMNs can be characterized as to their degrees of activation and functional capacity by way of identifying the expression of several activation markers on their surfaces known as clusters of differentiation (CD) glycoproteins [16,17]. The level of expression of these CD markers (e.g., CD11b, CD18, CD63, CD64, CD14, CD66a) identifies different PMN phenotypes associated with periodontal health and disease states [16], with oPMNs in the latter showing elevated degranulation, phagocytosis, reactive oxygen species production, neutrophil extracellular traps formation and expression of CD markers [16].

Human milk is a complex fluid that contains key nutritional components that are essential to the infant’s wellbeing and immune system development [18,19]. Of the cellular components found in human milk, PMNs (hmPMNs) are one of the main competent cells present, constituting 12–27% of the total population of leukocytes in milk [20]. The presence of leukocytes in human milk helps the infant to develop a functional immune system throughout childhood [21]. However, few studies have focused on the functionality of hmPMNs that can be related to low adherence, orientation, and motility, as compared to blood PMNs [21]. How hmPMNs activation states, illustrated by the expression levels of the CD markers, differ from those expressed on oPMNs remains unknown. Human milk also contains lipids and FAs [18,21], which are major sources of energy and are important precursors of essential metabolic compounds such as prostaglandins and thromboxanes [19]. Long-chain polyunsaturated fatty acids (LCPUFAs) are crucial nutrients in human milk and of particular importance since neurological and immunological development in a growing breastfeeding infant relies on the physiological actions of these FAs throughout childhood [22,23,24].

Evidence suggests that the abundance of human milk leukocytes including hmPMNs [25,26] and the concentration of FAs [27] change in response to the health status of the mothers and their infants. For example, elevated leukocyte counts occur during maternal infections (e.g., mastitis, cold, other organ-specific infections including eye, ear, vaginal, urinary tract and gastrointestinal infections) and/or infant infections (e.g., measles, upper respiratory infection, pneumonia, meningitis, acute otitis media, cold), and return to baseline levels upon recovery [25,26]. These findings suggest that there is a bidirectional relationship between maternal and infant health. Studies have also shown that the lipid content in human milk varies in the presence of several maternal health conditions, including but not limited to diabetes [28], obesity [29], and allergic diseases [30,31], which consequently affects the nutritional status and future health of the infant [32]. While studies have reported an association between maternal and infant infections and hmPMN counts and FAs content in human milk, the impact of maternal oral/periodontal inflammation on human milk composition is unknown.

Variations in the components of human milk including immune cells, FAs, bacteria, and oligosaccharides are all likely to be interrelated [33,34]. A correlation between FAs and immune cells in human milk including PMNs, eosinophils and lymphocytes has been demonstrated previously [34]. However, little is known about the correlation between FAs and hmPMN levels or activation status, based on expression of CD markers, in human milk.

Therefore, in this study we have investigated how the activation state of oPMNs differs from that of hmPMNs, based on the expression levels of CD markers. We have also investigated how the OIL in mothers during the first 4 months postpartum impacts the immunological components and nutritional value of human milk including the hmPMN counts and their activation state, as well as the content of FAs in human milk, and we have also shown how the health status of the infant can be correlated to changes in hmPMN counts, activation state, and FAs content in human milk over the first 4 months of lactation. Further, we have investigated whether there is a relationship between hmPMN counts and their activation state along with the levels of FAs in human milk.

## 2. Materials and Methods

This prospective cohort study was approved by the Research Ethics Board at St. Michael’s Hospital (REB#: 20-123), the Faculty of Dentistry, University of Toronto Research Ethics Board (40270), and the Carleton University Research Ethics Board (117222). Before collecting any maternal data, all participants signed written informed consent. This study was prepared using items on the Strengthening the Reporting of Observational Studies in Epidemiology (STROBE) checklist as a guide [35].

### 2.1. Study Population and Patient Selection

Fifty breastfeeding mothers were recruited from the Department of Obstetrics and Gynaecology at St. Michael’s Hospital between February 2021 and July 2021. Those who met the following inclusion criteria were invited to participate in this study: (A) Women 18 years of age or older who could give written consent in English; (B) Breastfeeding mothers of singletons who were healthy full-term new-borns with 5 min Apgar scores of 7 to 10 and who were delivered vaginally at 37 or more weeks of gestation. Participants were excluded if any of the following criteria were met (A) Smokers (e.g., tobacco, vaping, marijuana, etc.), (B) Having systemic diseases that could affect immune function and PMN counts or response (e.g., HIV infection, haematological disorders including but not limited to cyclic neutropenia and agranulocytosis, (C) Having diabetes (type I-II, gestational diabetes); (D) Having documented infection (apart from oral), such as a urinary tract and/or upper respiratory infection and/or mastitis at the time of recruitment, (E) Having taken antibiotics at the time of recruitment; (F) Having delivered their babies via C-section, (G) Having delivered twins or multiple babies.

Study samples were collected from mothers once at baseline (2–4 weeks postpartum) and once at follow-up (4 months postpartum). At these timepoints a study kit containing all the supplies needed for samples collection along with an instruction sheet was mailed to the participants. A video explaining how to collect the samples was also emailed to the participants.

### 2.2. Maternal Demographic Characteristics and Dietary Intake

Data regarding the demographic and behavioural characteristics of the study participants were collected using a structured questionnaire. Postnatal information such as mothers’ post-pregnancy body mass index (BMI), infant feeding patterns, infant hospitalization, and the use of antibiotics was also collected at both timepoints. Data regarding the gestational age, infant sex, and infant birthweight were obtained from the patients’ medical records. Infant health status was collected from self-reported questionnaires completed by the participants at both timepoints and was imputed on the basis of whether infants had been given antibiotics, or infants who had been hospitalized for any reason, and/or infants who had been diagnosed with any medical condition (e.g., asthma and milk protein allergic colitis), at least 2 weeks or longer prior to collection of samples. An infant is considered infected/diseased if any of these conditions were experienced, as opposed to healthy infants who did not experience any of these medical/health conditions. Since the maternal diet can affect lipid and FAs content in human milk, participants were asked to complete a 24-h dietary recall questionnaire using the validated Canadian version of the Automated Self-Administered 24-h Recall (ASA24^TM^) [36] twice; once at 2–4 weeks and once at 4 months postpartum. Data related to the energy (kcal/day), total lipids (g/day) and fatty acids (g) intake of participants were reported in the study, since these are known to affect the FAs composition in human milk.

### 2.3. Oral Rinse Samples Collection and Processing

oPMNs were collected from oral rinses and quantified based on a previously established protocol [8,9,10,11]. Participants were asked to rinse their mouth 6 times with 5 mL of 0.9% saline solution. Rinsing was to last for 30 s each time and they were to expectorate into a 50 mL Falcon tube, with a 2 min waiting time between each rinse. Multiple rinses are used to ensure enough absolute counts of oPMNs within the overall rinse sample to represent the participants’ OIL [8,11,37]. The saliva samples collected thusly were then stored on ice at 4 °C to preserve cells until processing at the laboratory within 2–4 h following collection. At the laboratory, 3.3 mL of paraformaldehyde (PFA) (16%) was added to the 30 mL saliva samples. The samples were fixed at 4 °C for 15 min on ice, then split into two equal aliquots and transferred to 15 mL Falcon tubes. The samples were then washed with phosphate-buffered saline (PBS) solution and centrifuged for 10 min at 100 RCF (2000 RPM). Pellets were resuspended in 10 mL of cold PBS total and filtered through a 40 μm filter followed by rinsing with 5 mL of cold PBS. Samples were centrifuged for 10 min at 2000 RPM and resuspended in 1 mL of PBS to wash. The samples were centrifuged again for 5 min at 20,000 RPM and then resuspended in 1 mL of flow cytometry staining (FACS) buffer. From this suspension, 25 uL was added to a cell counter vial and quantified using a Coulter counter.

The study samples were then categorized into 3 groups according to the absolute OIL: Healthy group “mothers with normal/low OIL”: oPMN counts <0.5 × 10^6^/10 mL rinse, Moderate group “mothers with moderate OIL”: oPMN counts 0.5–1 × 10^6^/10 mL rinse, and Severe group “mothers with severe OIL”: oPMN counts >1 × 10^6^/10 mL rinse. Participants were categorized in these groups based on the cut-offs reported in the literature to be correlated clinically with the various states of periodontal health and disease [8,10,38].

### 2.4. Human Milk Samples Collection and Processing

Before collecting milk samples, participants were asked to clean the breast (right and/or left, as preferred by the participant) with an alcohol swab or soap and water to reduce the likelihood of contamination of the samples by bacteria or nipple creams amongst other potential contaminants. Mothers were asked to completely express their milk manually or by use of a breast pump from one breast. Fifteen mL of human milk was added into the sterile 100 mL container provided in the kit. All mothers were asked to collect milk sample in the morning to standardize the collection method [39]. In addition, mothers were told that the amount of time elapsed since the last feeding on the breast, prior to obtaining the pumped or expressed samples, should be at least two hours [39]. Samples were then delivered to the laboratory on ice within 2–4 h of collection for analysis. The milk samples (10 mL) were analysed for the presence of hmPMNs using the same protocol as used for the oral rinse samples described above.

### 2.5. Flow Cytometry

Sample fixation, labelling, and flow cytometric analysis of oral rinse and milk samples were performed as described previously [16]. Oral and human milk PMNs were labelled with a panel of antibodies that could detect specific CD markers. The markers were classified into 4 categories based on function: degranulation/activation markers (CD63, CD64, and CD66a), immunoregulation markers (CD16), adhesion markers (CD11b, CD18), and lipopolysaccharide (LPS) receptor (CD14). Cells were labelled for 30 min on ice and in the dark before being washed 3 times with FACS buffer. For each CD marker, appropriate fluorescently tagged isotype control antibodies were used to determine auto fluorescent signals which were subtracted from mean fluorescence intensities (MFIs). Flow cytometer channel voltages were calibrated manually with rainbow beads to normalize sample acquisition on different days. Compensation was performed with single-stained OneComp eBeads (eBioscience, Waltham, MA, USA). Oral and human milk PMNs were gated based on high CD16 expression in combination with side scatter area (CD16^hi^ /SSC-A) (Figure S1). Sample acquisition was performed using a SONY spectral flow cytometer. Analysis was completed with FlowJo 10.0.7 (Tree Star; Ashland, OR, USA) and MFIs were determined for each CD marker.

### 2.6. Cell Sorting, Cytospin Preparation and Staining

Cells were sorted at a concentration of 10^6^ cells/mL in PBS/25% BSA (Flow buffer) using a FACSAria cell sorter (BD Biosciences, Franklin Lakes, NJ, USA). In order to confirm the purity of neutrophils, sorted cells were cytocentrifuged for 5 min at 800 RPM using a Cytospin centrifuge (Thermo Shandon, Waltham, MA, USA) as previously described, and then labelled using DiffQuik staining. Cell pellets were smeared onto slides so that bright field images could be acquired using a Nikon E1000 microscope with a 1003/1.3 oil immersion Plan Fluor objective (Nikon).

### 2.7. Fatty Acids Analysis in Human Milk

Gas chromatography flame ionization detection (GC-FID) was performed to identify and quantify FAs. Lipids were extracted from human milk following methodologies adapted from Folch et al. [40]. Briefly, 300 μL of human milk were homogenised and extracted in 4:2:1.75 chloroform– methanol–potassium chloride (0.88%) with 50 μg of C22:3n-3 used as an internal standard (NuChek-Prep, Elysian, MN, USA) as described previously [41]. Extracted samples were then to be dried down under nitrogen gas and then methylated by heating at 100 °C in 1:1 hexane–boron trifluoride methanol (Sigma 5 Aldrich, St. Louis, MO, USA). Deionised water was then added to stop the methylation reaction. Next, hexane was added to isolate the fatty acid methyl esters (FAMEs). FAMEs were quantified according to previously published methods using a Varian-430 gas chromatograph (Varian, Seattle, WA, USA). A quantity of 1 μL of sample was injected (splitless mode) into a capillary column (Agilent DB-23, Santa Clara, CA, USA; 30 m × 0.25 mm internal diameter, 0.25 μm film thickness) used to separate the FAMEs. A temperature of 250 °C was used for both the injector and detector. The oven temperature was initially set at 50 °C for 2 min, then increased at 20 °C/min until a temperature of 170 °C was reached. After 1 min at 170 °C, the temperature was increased by 3 °C/min until a final temperature of 212 °C was reached and held for 5 min. Helium was used as the carrier gas (0.7 mL/min). Identification of absorbance peaks was conducted using certified FAMEs standards (Nu-Chek Prep, Inc., Elysian, MN, USA). FAMEs peak analysis was conducted using Compass CDS software (version 3.0.0.68; Scion Instruments, Goes, NL) by proportional comparisons with the C22:3n-3 internal standard.

### 2.8. Statistical Analysis and Sample Size Estimate

The sample size calculation was based on previously published human milk studies that had focused on the concentration of PMNs in the human milk of mothers of infants with and without infection [26]. We determined that a sample size of 46 participants would provide a power of at least 80% to yield statistically significant results at *p* ≤ 0.05. The sample size was then increased to 50 participants to account for as-yet unknown variations in the OIL of these patients and for losses to follow-up.

Statistical analyses were performed using the SPSS statistical software (version 26.0, Armonk, NY, USA). The Kolmogorov–Smirnov normality test was used to confirm that the data were distributed normally in order to allow for the use of appropriate statistical tests. The primary exposure variable was defined as the three categorical OIL groups described above and which had been stratified based on absolute oPMN counts at baseline and follow-up. The primary outcome variables included hmPMN counts, the levels of their expressed CD markers, and the human milk FAs content (i.e., continuous). Continuous variables are presented as means and standard deviations (SD). We performed statistical analyses according to each of the study objectives. For the first objective, the paired samples *t*-test (for normally distributed data) and Wilcoxon Signed-Ranks test (for non-normally distributed data) were used to compare the activation states of oPMNs and hmPMNs at baseline and follow-up separately. Secondly, to compare the hmPMN counts, activation states, and the FA concentrations across the three OIL groups, the independent samples Kruskal–Wallis test (for non-parametric data) and one-way analysis of variance (ANOVA; for normally distributed data) (where the Tukey post-hoc test was used to evaluate if the means of the dependent variables were different across the three OIL groups) were performed for baseline and follow-up timepoints separately. *p*-values are presented for both unadjusted (from ANOVA/Kruskal-Wallis) and adjusted analyses. Adjusted analyses of the association between the exposure variable (i.e., three categorical OIL groups) and the outcome variables were also performed using multiple linear regression, and the following variables were included in the model as potential confounders; maternal age, post-pregnancy BMI, infant feeding pattern, infant sex, and oral health behaviours of mothers.

In order to compare the evolution of hmPMNs and their expressed CD markers, as well as the content of FAs during lactation (i.e., from baseline to follow-up timepoints) within each of the healthy and moderate/severe (defined as “Diseased” OIL group) OIL groups, linear mixed models were used using the participant ID as a random effect to account for repeated measures within mothers. For this analysis, moderate and severe OIL groups were grouped into one “Diseased” OIL group. This was done in order to increase the power of the study and be able to identify significant differences between the OIL groups during lactation, if there are any. By grouping moderate and severe OIL groups, we were able to identify those mothers with diseased OIL at both timepoints and compare them to mothers with healthy OIL at both timepoints. Data are presented as unadjusted and adjusted linear regression coefficients (β) along with the 95% confidence intervals (CIs) reported for mothers with diseased OIL, with mothers with healthy OIL as the reference group. The unadjusted coefficients correspond to the interaction term of OIL groups and time in order to determine whether the changes in the outcome variables over lactation are significantly different between the healthy and diseased OIL groups. The adjusted coefficients are presented after adjusting for the following confounding variables: maternal age and post-pregnancy BMI, infant feeding pattern, and oral health behaviours of mothers (EPA dietary intake was an additional confounding variable that was adjusted for in the models where FA levels were considered the outcome variables).

A linear mixed model was also used to evaluate the correlation between the infant health status and changes in hmPMN counts along with their expressed CD markers, and FAs composition in human milk during lactation considering both timepoints (i.e., exposure and outcomes variables at both baseline and follow-up). The exposure variable was defined as infants with and without health conditions at both timepoints (i.e., categorical). Unadjusted and adjusted linear regression coefficients (β) with 95% CIs were reported for infants who had a health condition (healthy infants were the reference group). In the linear mixed model, the unadjusted coefficients were based on time, exposure variable (i.e., infant health status), and their interaction, whereas the following potential maternal and foetal confounders were controlled for in the adjusted coefficients, maternal age and post-pregnancy BMI, infant feeding pattern, and infant sex. Linear mixed models were used in this study since they have greater power with continuous outcomes than linear regression models. Since time is continuously modelled, the power for detecting time-dependent effects increases [42]. Mixed models can also account for inter- and intra- individual differences and estimate predictor-dependent long-term changes in outcome variables by incorporating interaction terms between each variable in the model and time [43].

Finally, correlations between hmPMNs, CD biomarkers and FAs content were determined by Spearman’s rank correlation coefficients at each time point to address the study’s secondary objective. Statistical significance was set at *p* ≤ 0.05.

## 3. Results

### 3.1. Participants’ Descriptive Characteristics

Maternal demographic features and oral health characteristics at baseline are presented in Table 1. Study participants had a mean ± SD age of 34.5 ± 4.4 years, a BMI of 25.1 ± 3.7 kg/m^2^, and a gestational age of 39.1 ± 1.2 weeks. The majority of the participants were married (85.1%), employed full-time (68.1%), and had completed post-graduate studies (51.1%). The majority of participants (68.1%) reported brushing their teeth at least twice a day; however, only 25.1% reported flossing at least once daily. Regular dental visits (more than once a year) were reported by 80.9% of participants, and only 8.5% reported seeing a dentist only for emergencies. More than half of the participants also reported a dental visit within the previous year (57.4%). A summary score for overall maternal oral health behaviour was prepared and used in the adjusted model. The summary score was derived from four variables: participants’ tooth brushing frequency (0 = no brushing twice a day, 1 = brushing twice a day), flossing frequency (0 = no flossing once a day, 1 = flossing once a day), last dental visit (0 = last dental visit >1 year, 1 = last dental visit <1 year, and dental visit frequency (0 = dental visits only for emergency, 1 = ≤ 1 year, 2 = ≥ 1 year). For each variable, responses were coded as 0 and 1, except for the dental visit frequency, which was coded as 0, 1, and 2. Lower scores represent poorer oral health behaviours. Sixty-four percent of participants exclusively breastfed their infants, 22% predominantly breastfed their infants, and 8% used both human milk and formula equally.

### 3.2. Participants’ Oral Inflammatory Load

Out of the 50 mothers who participated in the study at baseline, 46 mothers were available for follow-up at 4 months postpartum (Figure S2). The absolute oPMN counts of the healthy, moderate, and severe groups at baseline and follow-up are presented in Table 2. At baseline, the mean ± SD of oPMN counts were 0.25 ± 0.15, 0.76 ± 0.14, and 2.66 ± 1.89 cells (×10^6^/10 mL rinse) in the healthy, moderate, and severe OIL groups, respectively. At follow-up, the mean ± SD of oPMN counts increased in the severe OIL group to 4.59 ± 6.38 (×10^6^ cells/10 mL rinse). A significant positive correlation between oPMN counts at base line and follow-up was found between oPMN counts at baseline and oPMN counts at follow-up (Spearman test, r_2_ = 0.352, *p* = 0.016).

### 3.3. Maternal Dietary Intake

Out of the 50 study participants, 40 and 42 completed the diet recall questionnaires at baseline and follow-up, respectively. Maternal reported intake of total lipids and FAs are shown in Table S1. At baseline, no differences were found in dietary intake according to maternal OIL, however at follow-up, mothers with severe OIL had significantly lower intake of eicosapentaenoic acid (EPA), compared to mothers with healthy and moderate OIL.

### 3.4. hmPMNs Had Lower Activation State Compared to oPMNs

**Figure 1:**
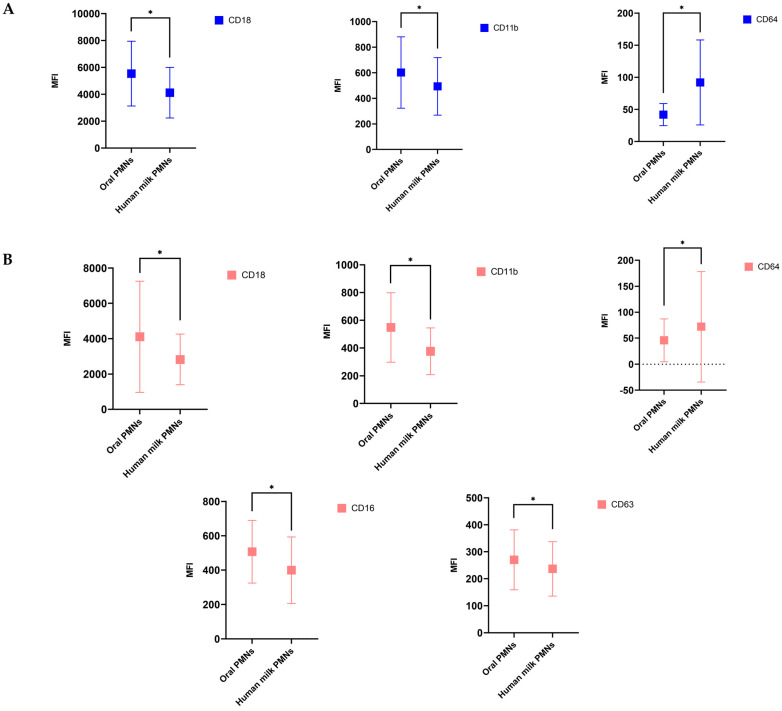
Cluster of differentiation (CD) biomarkers expression levels on human milk PMNs (hmPMNs) compared to oPMNs at baseline (**A**) and follow-up (**B**). At baseline (**A**), hmPMNs exhibited lower expression of CD18 and CD11b biomarkers and higher expression of CD64 biomarker compared to oPMNs. In contrast, at follow-up (**B**), hmPMNs exhibited lower expression of CD18, CD11b, CD16, and CD63 biomarkers compared to oPMNs. CD64 biomarker expressed on the hmPMNs was also upregulated significantly compared to oPMNs at follow-up. * denotes *p*-value ≤ 0.05. Data are presented as Mean ± SD with *p*-values from paired samples *t*-test (for normally distributed data) and Wilcoxon Signed-Ranks test (for non-normally distributed data) to compare the activation states of oPMNs and hmPMNs.

At baseline, hmPMNs exhibited lower expression of activation CD adhesion markers (CD11b and CD18) (*p* ≤ 0.05) and higher expression of the Fc gamma receptor 1 (CD64) (*p* ≤ 0.01) compared to oPMNs (Figure 1, Table S2). At follow-up, hmPMNs exhibited lower expression of activation of CD16 (immunoregulation marker) (*p* ≤ 0.01) and CD63 (degranulation/activation marker) (*p* = 0.042), in addition to the lower expression of CD11b and CD18 (adhesion markers) compared to oPMNs (Figure 1, Table S2). The Fc gamma receptor 1 (CD64) expressed on the hmPMNs was also upregulated significantly compared to oPMNs at follow-up (*p* ≤ 0.01) (Figure 1, Table S2).

### 3.5. Mothers with Moderate to Severe OIL Had hmPMNs with Higher Expression Levels of CD14 Biomarker and Lower Expression Levels of CD64 Biomarker during Lactation, Compared to Mothers with Healthy OIL

**Figure 2:**
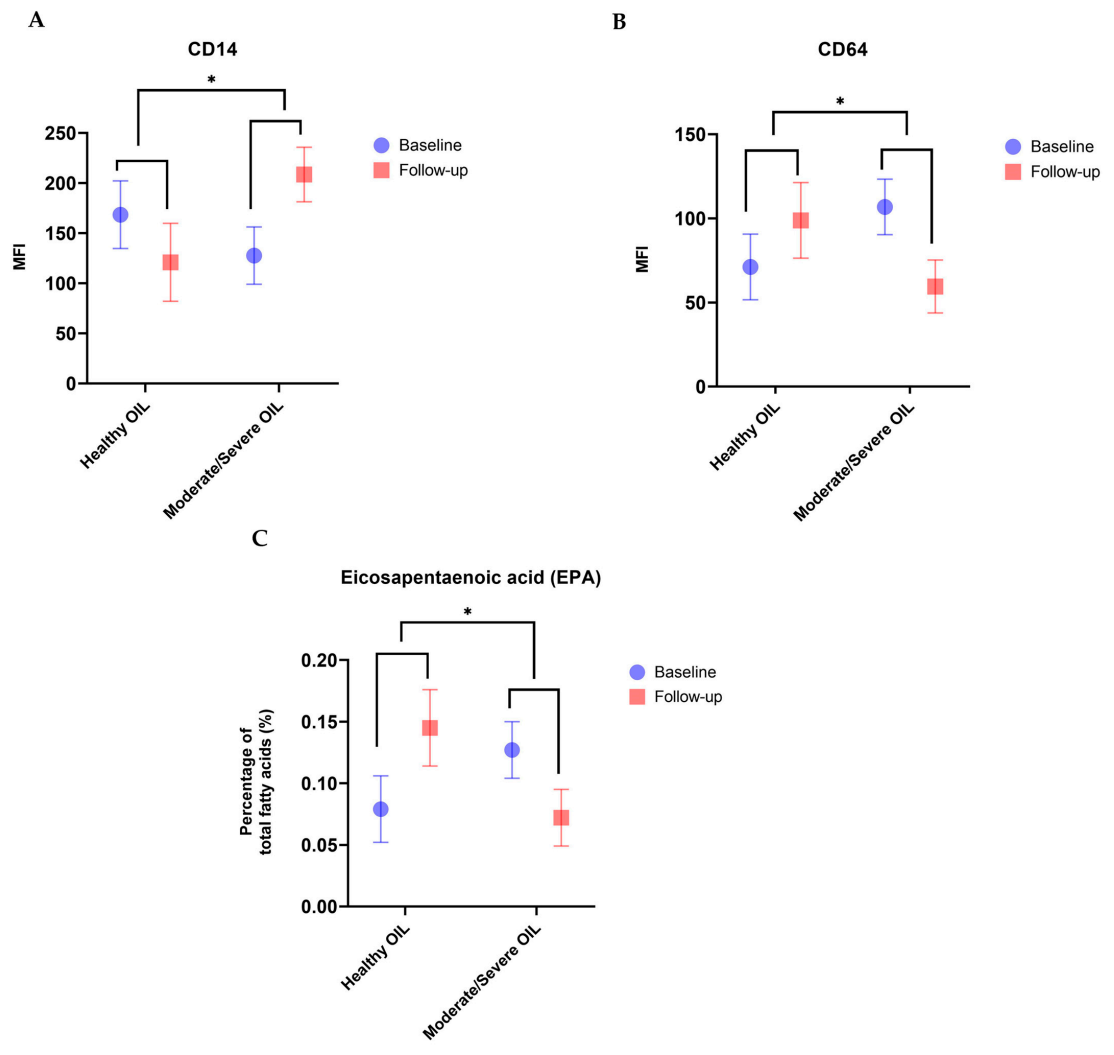
Differences in CD14 (**A**) and CD64 (**B**) expression levels and levels of n-3 poly-unsaturated fatty acid (PUFA); eicosapentaenoic acid (EPA) (**C**) between mothers with moderate/severe OIL and mothers with healthy OIL during lactation. Mothers with moderate and/or severe OIL at baseline and follow-up showed higher expression levels of CD14 (**A**) and lower expression levels of CD64 (**B**) biomarkers and lower levels of EPA n-3 PUFA (**C**) at follow-up compared to baseline, compared to mothers with healthy OIL. * denotes *p*-value ≤ 0.05. Data are presented as Mean ± SD.

There were no statistically significant differences in hmPMN counts and activation states between mothers with healthy, moderate, and severe OIL at both baseline and follow-up, separately (Table S3). Furthermore, no differences in the hmPMN counts and activation states were observed when moderate and severe groups (as a “Diseased” OIL group) were combined and compared with mothers with healthy OIL (data not shown). When assessing the differences in the absolute hmPMN counts and activation states during lactation between mothers with diseased OIL and mothers with healthy OIL, we found that among mothers with diseased OIL, there was a statistically significant decrease in the expression of CD64 biomarker (β = −95.75, adjusted *p*-value = 0.043; Figure 2, Table 3), and a statistically significant increase in the expression of CD14 biomarker (β = 151.25, adjusted *p*-value = 0.031; Figure 2, Table 3) over the first 4 months of lactation, compared to mothers with healthy OIL, after adjusting for potential confounders. Changes in the absolute hmPMN counts and expression levels of CD16, CD66a, CD11b, CD18, and CD63 did not differ significantly between mothers with healthy and diseased OIL during lactation (*p* > 0.05; Table 3).

### 3.6. Mothers with Moderate and Severe OIL Had Higher Levels of n-6 PUFA; Docosapentaenoic Acid (DPA) in Their Human Milk at Baseline, Compared to Mothers with Healthy OIL

At baseline, the levels of SFAs, MUFAs, and PUFAs were similar across the OIL groups. However, mothers with moderate and severe OIL had significantly higher levels of C22:5n-6 (n-6 PUFA; docosapentaenoic acid (DPA)) in their human milk compared to mothers with healthy OIL (*p* = 0.036; Table S4). Furthermore, after controlling for confounders, mothers with moderate OIL had significantly higher levels of C22:5n-6 and C22:4n-6 at baseline compared to healthy mothers (*p* < 0.05; Table S4), but this association was not significant for the severe OIL group in the adjusted model. At follow-up, mothers with severe OIL tended to have the highest n6:n3 ratio compared to mothers with healthy and moderate OIL; however, these differences were not statistically significant in adjusted and unadjusted models (*p* > 0.05; Table S4). Overall, levels of fatty acids at follow-up were similar across the OIL groups.

### 3.7. Mothers with Moderate to Severe OIL Had Lower Levels of C20:5n-3 (EPA) LCPUFA during Lactation, Compared to Mothers with Healthy OIL

After assessing the differences in the FA levels during lactation between mothers with healthy and diseased OIL, we found that human milk of mothers with moderate to severe (i.e., diseased) OIL showed lower levels of C20:5n-3 EPA LCPUFA (β = −0.11, *p* = 0.029; Figure 2, Table 4) during lactation compared to mothers with healthy OIL, and this difference reached borderline significance after adjusting for potential confounders (β = −0.12, *p* = 0.056; Figure 2, Table 4). However, there were no statistically significant differences in the levels of the other FAs between mothers with healthy and diseased OIL (*p* > 0.05; Table 4).

### 3.8. CD64 Surface Expression on hmPMNs Was Higher in Mothers Whose Infants Had a Health Condition Compared to the Mothers of Healthy Infants

Infant health status was associated significantly with changes in only the CD64 activation biomarker on the hmPMNs, where CD64 expression was upregulated on hmPMNs from the mothers of infants with any health complication (β: 85.65, *p* = 0.009 in adjusted models; Table 5, Figure S3). There were no significant associations between the infant health status, the absolute hmPMN counts, and the other CD biomarkers (*p* > 0.05; Table 5).

### 3.9. Higher Levels of Arachidonic Acid and C22:5n-6 FA Were Observed in the Human Milk of the Mothers Whose Infants Had a Health Condition Compared to Healthy Infants

Infant health status was shown to be associated significantly with changes in the levels of C24:0, C20:4n-6, C22:5n-6 (*p* < 0.05; Table 6). Compared to healthy infants, the human milk of mothers whose infants had a health condition or infection had significantly higher concentrations of C24:0 (β: 0.03, *p* = 0.004), C20:4n-6 (β: 0.12, *p* = 0.013), and C22:5n-6 (β: 0.06, *p* = 0.015) FAs, after adjusting for confounding variables (Table 6). There were no significant associations between the infant health status and the other FAs (*p* > 0.05; Table 6).

### 3.10. Correlation between Human Milk PMNs and Their Levels of Activation and the FAs in Human Milk

**Figure 3:**
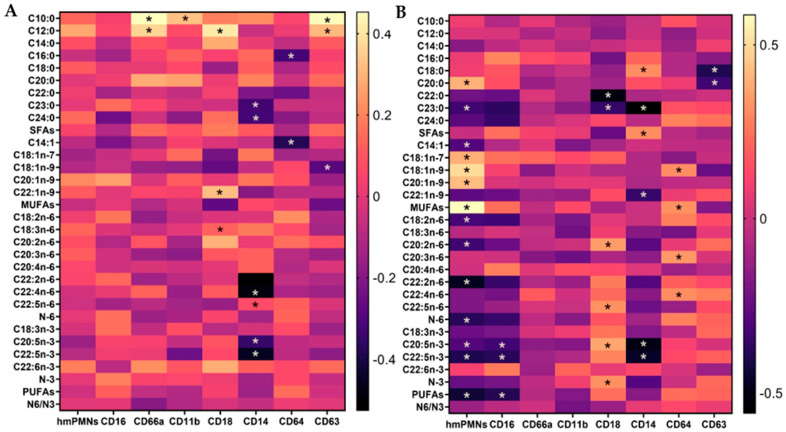
Heat map of correlation coefficients between human milk neutrophils (hmPMNs) and expression of CD markers, along with the fatty acids (FAs) content in human milk at baseline (**A**) and follow-up (**B**). The rows represent the FAs in human milk. Columns represents the hmPMNs and CD markers expression levels. The range of values is represented by the colour key. * denotes *p*-value ≤ 0.05.

At baseline, there was no observed correlation between human milk PMNs, the CD16 biomarker, and the FAs in human milk (*p* > 0.05; Figure 3, Table S5). A significant moderate positive correlation with CD66a was shown for C10:0 and C12:0 FAs (*p* < 0.05; Figure 3, Table S5). Furthermore, higher expression levels of CD14 biomarker were associated with lower levels of n-3 and n-6 PUFAs. Specifically, a moderate negative correlation between CD14 biomarker and C22:2n-6, C22:4n-6, C20:5n-3, and C22:5n-3 FAs was demonstrated (*p* < 0.05; Figure 3, Table S5). CD64 and CD63 were negatively correlated with C14:1 and C18:1n-9, respectively (*p* < 0.05; Figure 3, Table S5).

As opposed to what was found at baseline, higher levels of hmPMNs were associated with higher levels of some SFAs and MUFAs, where a significant but moderate positive correlation was observed between hmPMN counts and C20:0, C18:1n-7, C18:1n-9, C20:1n-9, and MUFAs. However, higher levels of hmPMNs were associated with lower levels of n-3 and n-6 PUFAs. Specifically, a significantly moderate negative correlation between hmPMN counts and C18:2n-6, C20:2n-6, C22:2n-6, N-6, C20:5n-3, C22:5n-3, and PUFAs at follow-up was observed (*p* < 0.05; Figure 3, Table S6). Higher expression levels of CD16 biomarker were associated with lower levels of n-3 and n-6 PUFAs, where CD16 biomarker was negatively correlated with C23:0, C24:0, C18:2n-6, C22:2n-6, N-6, C20:5n-3, C22:5n-3, and PUFAs (*p* < 0.05; Figure 3, Table S6). CD664a and CD11b biomarkers did not correlate with C10:0, C12:0 FAs in human milk, as opposed to the baseline (*p* > 0.05; Figure 3, Table S6). Compared to the baseline, similar correlations were observed at follow-up, where CD18 showed a moderate positive correlation with C20:2n-6 at follow-up (*p* < 0.05; Figure 3, Table S6). Furthermore, CD14 was negatively correlated with C23:0, C22:1n-9, C20:5n-3, and C22:5n-3 at follow-up, same as baseline (*p* ≤ 0.05; Figure 3, Table S6).

## 4. Discussion

In this study, hmPMNs were shown to have lower activation state compared to hmPMNs at baseline and follow-up. This study also showed that moderate to severe OIL is associated with changes in the activation state of hmPMNs and the concentrations of FAs in human milk. Significant associations between the status of infant health, the CD markers expressed on hmPMNs and the composition of FAs in human milk were also suggested. Moreover, this study investigated correlations between activation levels/expression of CD markers on hmPMNs with FAs in human milk.

In this study, we reported new findings regarding the differences in the activation states between PMNs found in the oral cavity and human milk (Figure S4). This difference in the activation states between hmPMNs and oPMNs might be due to the shedding of these molecules/markers when they move from the blood and pass through the mammary gland and into human milk [44]. Studies also reported that human milk contains inhibitors which affect the activity and function of PMNs during lactation [18,45], since it has been reported that in human milk, PMNs ingest fat globules and casein micelles resulting in the loss of their cytoplasmic granules and altering the PMNs phagocytic capacity and bactericidal activities [45,46]. However, CD64 biomarker was elevated on hmPMNs compared to oPMNs at both timepoints, suggesting that there might be a unique role for the high-affinity Fc-receptor, CD64, on hmPMNs, for example in strengthening antibody-mediated protection in the new-born infant. The expression of the CD64 marker on blood PMNs is being investigated as a potential screening marker for the detection of infection in infants [47]. Therefore, the CD64 biomarker, being expressed highly on hmPMNs compared to oPMNs can point to a potentially crucial role in fighting infections in neonates.

Another finding in this study is that mothers with moderate to severe OIL had higher expression levels of CD14 biomarker and lower expression levels of CD64 biomarker on their hmPMNs during lactation, compared to mothers with healthy OIL (Figure S5). This change in the expression levels might be related to the presence of pathogenic gram-negative anaerobic microorganisms of periodontal diseases in human milk such as *Porphyromonas gingivalis* [48]. CD14 biomarker expressed on PMNs acts as a receptor for the LPS of gram-negative bacteria and studies reported higher expression levels of CD14 on oPMNs of patients with periodontitis compared to healthy individuals [49]. Since the pathogenicity of gram-negative anaerobic bacteria of periodontal diseases lie in its endotoxins including LPS [49], therefore it can be hypothesized that the higher expression levels of CD14 on hmPMNs of mothers with moderate to severe OIL might be related to the presence of these pathogenic bacteria in their human milk. Furthermore, studies have shown that the virulence factors of pathogenic bacteria can interfere with the neutrophils’ bactericidal activities, chemotaxis, and inhibition of neutrophil phagocytosis, where recent studies have shown that the upregulation of CD64 biomarker was inhibited when exposed to pathogenic oral bacteria, as opposed to its upregulation in case of commensal bacteria [48]. Therefore, given the downregulation of CD64 biomarker on hmPMNs of mothers with moderate to severe OIL, it might again be hypothesized that this can be due to the presence of gram-negative anaerobic bacteria in their human milk. Future studies that focus on the colonization of these pathogenic bacteria in human milk are much needed to confirm such hypothesis. This state of downregulation of CD64 biomarker on hmPMNs might negatively impact the functional capacity of hmPMNs when transferred to the breastfed infants, and consequently affect the ability of hmPMNs to adhere to or migrate to the alimentary tract mucosa of the infants [46]. To our knowledge, no studies have previously investigated the impact of maternal infections on the activation state of hmPMNs. Therefore, more longitudinal studies are needed to understand more clearly, the impact of maternal infections on the activation state of hmPMNs and how this can affect infant health outcomes earlier and later in their life.

With respect to the FAs composition in human milk, mothers with moderate to severe OIL had elevated levels of n-6 DPA compared to mothers with healthy OIL at baseline. Given the proinflammatory nature of n-6 PUFAs, the elevated levels of n-6 DPA in the milk of mothers with moderate to severe OIL might suggest an inflammatory response in the mammary gland triggered by oral inflammation in mothers. This parallels results reported in a recent systematic review by Peila et al. [28] showing higher concentrations of four essential n-6 PUFAs (ALA, eicosatrienoic acid, AA and docosatetraenoic acid) in the colostrum of diabetic mothers, compared to their non-diabetic counterparts. However, by analysing the evolutional changes in human milk in correlation to each OIL group (healthy vs. moderate/severe groups), we found that the concentrations of EPA, one of the essential n-3 LCPUFAs, decreased in the milk of mothers with moderate to severe OIL during lactation compared to mothers with healthy OIL after adjusting for confounding variables. Regarding the association between maternal chronic diseases and the FA composition of human milk, studies in the literature have investigated the possible influence of elevated body weight, diabetes mellitus, allergic diseases, and arterial hypertension on the FA content of human milk [32]. In as much as the former conditions can be considered inflammatory in nature, the findings reported here comport with those reported in the previous studies. The latter demonstrated low concentrations of PUFAs (ALA and DHA) in the human milk of obese mothers, compared to those with normal weight [29,50]. This is also consistent with findings from studies that demonstrated lower levels of PUFAs (EPA, DHA and DPA) in HIV-infected mothers compared to their HIV-uninfected counterparts [51].

N-6 and n-3 PUFAs have proinflammatory and anti-inflammatory properties, respectively, [52,53,54]. In general, FAs in human milk can be produced by either de novo fatty acid synthesis in the mammary gland, mobilized from maternal adipose tissues, or directly supplied by the maternal dietary intake [55]. This difference in the EPA levels in the current study are not explained by maternal dietary intake, since there were no differences in the dietary intake of PUFAs between the study groups at both timepoints, and difference in the EPA dietary intake between the study groups at follow-up was accounted for in the adjusted analyses. A possible hypothesis of the low levels of EPA in the human milk of mothers with moderate to severe OIL might be as a result of the overall systemic inflammatory load caused by periodontal diseases causing the over production of pro-inflammatory mediators that can enter the bloodstream together with the bacterial endotoxins [1]. This state of inflammation might consequently alter the function of mammary gland cells interfering with or delaying lactogenesis and ultimately affecting the FAs content in human milk [30,31]. Another hypothesis might be that the mammary glands of mothers with moderate to severe OIL might have a reduced capacity for conversion and uptake of FAs from circulation into lipids found in human milk [51]. EPA is one of the three major LCPUFAs found in the brain and retina, and studies show that the neurological and immunological developments of the infant throughout childhood rely significantly on these FAs [23]. Although the clinical implications of lower levels of EPA are still unclear, studies in the literature support the idea that this might impair neurodevelopment in infants because of problems relating to the transfer of essential PUFAs required to meet the specific infant’s needs, particularly in the first months of their life [56].

Another finding in this study was the higher expression of CD64 biomarker on hmPMNs in mothers whose infants had a health condition, compared to the mothers of healthy infants. Recently there has been an increased interest in investigating CD64 biomarker expression on the blood PMNs of neonates to screen for neonatal sepsis [47,57,58,59]. Ng et al. [59] reported higher expression of neutrophil CD64 marker in the blood of infected infants (bacterial infections and pneumonia) compared to non-infected infants. CD64 biomarker plays an important role in the phagocytosis and intracellular killing of bacteria [47,57,59], and is expressed at low levels on non-activated PMNs without bacterial stimulation [56]. However, at the onset of any infection, CD64 becomes upregulated and expressed at significantly higher concentrations [47,58]. A possible explanation of the upregulation of the CD64 biomarker on hmPMNs could be that the CD64 on the hmPMNs might be able to recognize the bacteria affecting infants through the transmission of infection from the oral cavity of infected infants to mothers through breastfeeding and reaching the human milk environment [60]. As a protective mechanism [61], CD64 on hmPMNs is upregulated to improve the PMNs’ phagocytosis functions to be able to fight infant infections. Therefore, future studies investigating the diagnostic accuracy of CD64 marker on hmPMNs for sepsis and infections in neonates are needed. Higher levels of n-6 LCPUFAs including AA and C22:5n-6 were also observed in the human milk from mothers of infants who had a health condition, compared to human milk from mothers of healthy infants. As mentioned above, n-6 PUFAs are pro-inflammatory in nature and play a critical role in the inflammatory immune response [22,62], particularly AA, which is essential for the development and functionality of the infant immune system since it acts as a precursor to a number of eicosanoids (e.g., leukotrienes, prostaglandins, thromboxanes) that play an important role in regulating different immune responses [62,63]. Therefore, the high levels of AA in the human milk of mothers of infected infants might be an inflammatory response mechanism triggered by the infant’s infection. This is believed to be a beneficial response so the infant’s immune system can fight the infection [62,63]. However, further research is warranted to identify the effect of elevated AA levels on infant health in the short and long term.

This study showed moderate negative correlations between hmPMNs counts and PUFAs in human milk at follow-up. However, these findings were not consistent with our hypothesis that higher counts of hmPMNs would be associated with higher levels of PUFAs, particularly n-6 PUFAs [64]. The observed negative correlation between hmPMNs and PUFAs may suggest a mechanism to create a ‘balanced immune response’ or could indicate a state of homeostasis in the mammary gland [34]. Moreover, the CD14 biomarker on hmPMNs was correlated negatively with n-3 PUFAs; EPA and C22:5n-3 at baseline and follow-up. CD14 biomarker is an LPS-binding protein that promotes the secretion of innate immune response molecules (e.g., interleukin-8, tumour necrosis factor-alpha) and thus plays a role in antimicrobial host defence [33,65]. Soluble CD14 protein is highly abundant in human milk and initiates an immune response in the infants’ gut in response to pathogens [33,65,66]. On the other hand, n-3 PUFAs have anti-inflammatory properties, which might explain the negative correlation with the CD14 biomarker. However, CD64 biomarker expressed on hmPMNs showed a moderate positive correlation with n-6 PUFAs; C22:4n-6 and C20:3n-6 at follow-up. As discussed above, CD64 is an essential biomarker for screening of sepsis in infants, so when highly expressed on hmPMNs, this might also promote the expression of n-6 PUFAs, which play a major proinflammatory role in helping infants fight any infections during the early few months of their life.

### Study Limitations and Strengths

This study has a number of limitations. First, the study population included only mothers served by St. Michael’s Hospital, limiting the study results’ generalizability. Second, nutritional, immunological, and other bioactive components in human milk change at each stage of lactation, making it challenging for human milk studies to identify the impact of specific exposure variables on these changes in human milk composition. That is why collecting human milk samples over a 24-h period is considered the gold standard method of collection; however, this method is not feasible and was not used in our study. Consequently, this could have provided variability in human milk components. However, to address this issue, all mothers collected human milk samples only in the morning for standardization purposes at both timepoints.

Moreover, with respect to the infants’ health status, mothers did not specify in the questionnaires the reason their infants took antibiotics and not all mothers specified why their infants were hospitalized, which makes the changes in CD markers and FA levels imperfect surrogates of infant health and cannot point to specific infant diseases or conditions. However, there still appears to be some association between health status, as defined in this investigation, and the other biological and biochemical markers studied here. Larger longitudinal studies are critically needed to assess the effect of specific infant infections using objective rather than subjective measures, on the composition of human milk. Another limitation is that only the BMI of participants after delivery was collected from the participants’ records, and any change in the BMI of participants at the 4-month follow-up timepoint was not recorded. Therefore, only BMI after delivery was controlled for in the adjusted analyses. Given that the BMI is one of the main factors that are known to affect human milk fatty acid composition [29,50], it is critically important that future studies collect BMI at each follow-up timepoint, to ensure that the exposure variable is correlated with any changes in the FA levels.

This study is strengthened by the data that illustrate, in a longitudinal manner, compositional changes in human milk. Since human milk samples were collected at two time points, this allowed for a reliable understanding of alterations in the content of FAs in human milk over the first few months of lactation. Furthermore, with the variation of milk composition over lactation, the individual differences in the outcome measures were assessed at both time points using the linear mixed model. Since FAs are considered critically important for the development of immunity and development in infants [22], the findings shown here point to the importance of understanding and possibly treatment of maternal oral diseases. This study also included dietary recall data from mothers at both time points, which add to its strengths and also demonstrated that maternal OIL increased at 4 months postpartum relative to baseline, indicating a worsening of oral health in mothers after childbirth. This is consistent with the fact that mothers can be at risk of poor oral health during the challenging postpartum period [4], and proper oral hygiene measures should be emphasized.

## 5. Conclusions

Human milk neutrophils have lower activation states compared to oral neutrophils. Mothers with moderate to severe OIL had hmPMNs that expressed higher expression levels of CD14 biomarker and lower expression levels of CD64 biomarker. Low levels of EPA were also observed in the human milk of mothers with moderate to severe OIL at follow-up, compared to baseline. The health status of infants might also affect human milk composition, characterized principally by elevation in the expression of CD64 biomarker on hmPMNs and elevation in the AA and C22:5n-6 levels in the human milk of mothers of infants with a health condition or infection, a finding that requires much more precise characterization. This study underscores the impact of both maternal oral health and infants’ general health on human milk composition. How these alterations can affect infant health outcomes in the long term critically need to be considered.

## Figures and Tables

**Table 1:** Participants’ demographics and oral health-related characteristics.

Characteristics	Variables	Total N (%)
Demographic characteristics	**Age (years)**	
	(Mean ± SD)	34.5 ± 4.4
	**Marital status**	
	Married	40 (85.1)
	Single/Unmarried partners	7 (14.9)
	**Employment status**	
	Employed full-time	32 (68.1)
	Employed part-time/Student/unemployed	15 (31.9)
	**Highest education level**	
	Secondary graduate/Degree/Diploma	23 (48.9)
	Post-graduate studies	24 (51.1)
Maternal and infant characteristics	**Gestational age at delivery (weeks)**	39.1 ± 1.2
	(Mean ± SD)	
	**BMI after delivery (kg/m^2^)**	25.1 ± 3.7
	(Mean ± SD)	
	**Infant feeding patterns**	
	Exclusive BF	32 (64.0)
	Predominant BF	11 (22.0)
	Mixed feeding *	4 (8.0)
	**Infant sex**	
	Boy	26 (52.0)
	Girl	22 (44.0)
	**Infant health status**	
	Healthy	39 (78.0)
	Diseased/Infected	8 (16.0)
Lifestyle and dental history	Brushing at least twice a day	32 (68.1)
	Flossing at least once per day	12 (25.1)
	**Dental visit frequency**	
	≥1 visit a year	38 (80.9)
	<1 visit a year	5 (10.6)
	Only for emergency	4 (8.5)

BMI: Body Mass Index, BF: Breastfeeding, *Mixed feeding is defined as infants receiving both breast milk and formula equally.

**Table 2:** Mean ± SD values of the oPMN counts (×10^6^ cells per 10 mL rinse) of the study population at baseline and follow-up.

oPMN Counts (×10^6^/10 mL Rinse)	N	Mean ± SD	Median	Minimum	Maximum
**Total oPMN counts**					
Baseline	50	1.38 ± 1.63	0.65	0	6.97
Follow-up	46	1.93 ± 4.24	0.72	0	23.56
**Baseline**					
Healthy	21	0.25 ± 0.15	0.35	0	0.47
Moderate	8	0.76 ± 0.14	0.65	0.52	0.98
Severe	21	2.66 ± 1.89	1.89	1.01	6.97
**Follow-up**					
Healthy	16	0.22 ± 0.15	0.29	0	0.49
Moderate	13	0.78 ± 0.13	0.7	0.5	0.93
Severe	17	4.59 ± 6.38	1.43	1.1	23.57

oPMN: oral polymorphonuclear neutrophils. SD: standard deviation.

**Table 3:** Comparison of hmPMN counts and CD biomarkers expression levels by maternal oral inflammatory load (OIL) status ^a^.

Neutrophils and CD Biomarkers ^†^	Diseased OIL (*n* = 20)			
	Estimate β ^b^ (95% CI)	*p*-Value	Adjusted Estimate β ^c^ (95% CI)	Adjusted *p*-Value
Absolute hmPMN counts (×10^6^/10 mL)	0.23 (−0.10, 0.57)	0.168	0.27 (−0.11, 0.65)	0.17
CD16	−175.72 (−419.60, 68.15)	0.156	−172.41 (−447.57, 102.75)	0.216
CD66a	−86.62 (−236.12, 62.88)	0.253	−76.77 (−270.10, 116.55)	0.431
CD11b	−6.26 (−175.29, 162.76)	0.941	17.21 (−171.75, 206.17)	0.856
CD18	445.38 (−991.48, 1882.25)	0.539	503.41 (−1000.33, 2007.16)	0.507
CD14	128.22 (−2.34, 258.80)	0.054	151.25 (14.13, 288.36)	**0.031**
CD64	−75.04 (−149.00, −1.07)	**0.047**	−95.35 (−187.69, −3.01)	**0.043**
CD63	−26.44 (−145.40, 92.51)	0.66	−4.58 (−133.71, 124.54)	0.944

^a^ Estimates are for mothers with “Diseased” OIL (i.e., mothers with moderate/severe OIL) as compared to mothers with “Healthy” OIL, who were defined as the reference group. ^b^ Unadjusted linear mixed model based on the terms time (i.e., stage of lactation), OIL categorized variable and the interaction of OIL groups * time. ^c^ Adjusted for maternal age and post-pregnancy BMI, infant’s feeding pattern, and oral health behaviour score of mothers in linear-mixed regression model. ^†^ For the CD markers, data are presented as the geometric mean fluorescence intensity (MFI) for each CD marker. CI: Confidence Interval.

**Table 4:** Comparison of fatty acid levels in human milk by maternal OIL status ^a^.

Fatty Acids in Human Milk	Diseased OIL (*n* = 20)			
	Estimate β ^b^ (95% CI)	*p*-Value	Adjusted Estimate β ^c^ (95% CI)	Adjusted *p*-Value
C10:0	−0.17 (−0.49, 0.14)	0.269	−0.15 (−0.50, 0.18)	0.368
C12:0	−0.80 (−2.06, 0.46)	0.210	−0.91 (−2.27, 0.44)	0.182
C14:0	−0.57 (−1.85, 0.71)	0.376	−0.50 (−1.80, 0.79)	0.440
C16:0	0.94 (−1.77, 3.65)	0.492	1.63 (−1.29, 4.57)	0.269
C18:0	1.27 (0.10, 2.43)	**0.032**	1.11 (−0.15, 2.39)	0.084
C20:0	0.01 (−0.02, 0.06)	0.439	0.01 (−0.04, 0.06)	0.739
C22:0	0.01 (−0.04, 0.05)	0.865	−0.01 (−0.06, 0.04)	0.674
C23:0	−0.03 (−0.29, 0.22)	0.772	−0.05 (−0.34, 0.24)	0.719
C24:0	0.01 (−0.03, 0.04)	0.731	0.01 (−0.03, 0.04)	0.799
**SFAs**	0.60 (−4.02, 5.22)	0.796	0.80 (−4.32, 5.93)	0.756
C14:1	−0.05 (−0.12, 0.02)	0.192	−0.01 (−0.09, 0.07)	0.795
C18:1n-7	−2.97 (−10.66, 4.72)	0.445	−1.83 (−10.75, 7.08)	0.683
C18:1n-9	4.21 (−4.43, 12.87)	0.335	2.98 (−6.76, 12.74)	0.543
C20:1n-9	0.05 (−0.03, 0.13)	0.240	0.03 (−0.05, 0.12)	0.469
C22:1n-9	−0.07 (−0.21, 0.05)	0.242	−0.11 (−0.26, 0.04)	0.151
**MUFAs**	1.43 (−3.17, 6.03)	0.539	1.18 (−3.64, 6.01)	0.627
C18:2n-6 (LA)	−1.22 (−6.74, 4.29)	0.661	−0.64 (−6.69, 5.39)	0.831
C18:3n-6 (GLA)	0.00 (−0.04, 0.04)	0.969	0.01 (−0.03, 0.04)	0.851
C20:2n-6	0.08 (−0.23, 0.39)	0.604	0.06 (−0.28, 0.42)	0.705
C20:3n-6	0.05 (−0.03, 0.15)	0.233	0.03 (−0.07, 0.14)	0.501
C20:4n-6 (AA)	0.02 (−0.09, 0.14)	0.687	−0.01 (−0.13, 0.12)	0.914
C22:2n-6	0.71 (−1.18, 2.61)	0.458	0.59 (−1.60, 2.80)	0.589
C22:4n-6	−0.08 (−0.21, 0.04)	0.188	−0.12 (−0.26, 0.01)	0.088
C22:5n-6	−0.05 (−0.12, 0.01)	0.075	−0.04 (−0.11, 0.02)	0.166
**N-6**	−1.10 (−7.38, 5.18)	0.729	−0.50 (−7.39, 6.38)	0.884
C18:3n-3 (ALA)	0.21 (−0.50, 0.93)	0.558	−0.06 (−0.85, 0.73)	0.880
C20:5n-3 (EPA)	−0.11 (−0.22, −0.01)	**0.029**	−0.12 (−0.24, 0.00)	**0.056**
C22:5n-3	−0.06 (−0.19, 0.06)	0.336	−0.11 (−0.25, 0.03)	0.132
C22:6n-3 (DHA)	−0.04 (−0.23, 0.14)	0.647	−0.06 (−0.27, 0.14)	0.536
**N-3**	−0.10 (−0.96, 0.75)	0.807	−0.42 (−1.36, 0.51)	0.369
**PUFAs**	−0.84 (−7.41, 5.72)	0.799	−0.48 (−7.62, 6.66)	0.894
**N6/N3**	4.33 (−7.19, 15.86)	0.457	5.52 (−7.54, 18.59)	0.402

^a^ Estimates are for mothers with “Diseased” OIL as compared to mothers with “Healthy” OIL, who were defined as the reference group. ^b^ Unadjusted linear mixed model based on the terms time (i.e., stage of lactation), OIL categorized variable and the interaction of OIL groups * time. ^c^ Adjusted for maternal age and post-pregnancy BMI, infant’s feeding pattern, oral health behaviour score of mothers, and eicosapentaenoic acid (EPA) maternal dietary intake in linear-mixed regression model. SFAs: saturated fatty acids; MUFAs: mono-unsaturated fatty acids; LA: linoleic acid; GLA: gamma-linolenic acid;AA: arachidonic acid; ALA: α-linolenic acid; EPA: eicosapentaenoic acid; DHA: docosahexaenoic acid; PUFAs: poly-unsaturated fatty acids.

**Table 5:** Significance evaluation of infants’ health status on the changes in CD64 biomarkers expression levels on hmPMNs over lactation.

Neutrophils and CD Markers	Infants with a Health Condition (*n* = 14) ^c^			
	Estimate β ^a^ (95% CI)	*p*-Value	Adjusted Estimate β ^b^ (95% CI)	Adjusted *p*-Value
**Absolute hmPMN counts (×10^6^/10 mL)**	−0.13 (−0.49, 0.21)	0.288	−0.05 (−0.30, 0.18)	0.624
**CD16**	−46.23 (−306.54, 214.07)	0.553	60.94 (−170.74, 292.63)	0.599
**CD66a**	−33.50 (−189.92, 122.91)	0.890	−36.03 (−155.85, 83.78)	0.547
**CD11b**	10.91 (−169.38, 191.21)	0.602	−26.22 (−142.45, 90.00)	0.650
**CD18**	861.03 (−635.55, 2357.63)	0.423	359.51 (−781.69, 1500.73)	0.529
**CD14**	81.35 (−51.31, 214.02)	0.392	38.05 (−37.85, 113.95)	0.316
**CD64**	100.25 (27.69, 172.80)	**0.019**	85.65 (21.97, 149.32)	**0.009**
**CD63**	−4.75 (−121.62, 112.11)	0.183	−54.96 (−150.39, 40.47)	0.250

^a^ Unadjusted linear mixed model based on the terms time (i.e., stage of lactation), infant health status variable and their interaction (Infants without a health condition were considered the reference group). ^b^ Adjusted for time, maternal age and BMI after delivery, infant’s feeding pattern and infant sex. ^c^ Out of the 14 infants, 10 were breastfed exclusively and 4 were predominantly breastfeeding. Two infants have been given antibiotics, 8 infants were hospitalized for any reason, and 4 infants were diagnosed with medical conditions (asthma and milk protein allergic colitis).

**Table 6:** Association between infant health status and human milk fatty acids levels during lactation.

Fatty Acids in Human Milk	Infants with a Health Condition (*n* = 14)			
	Estimate β ^a^ (95% CI)	*p*-Value	Adjusted Estimate β ^b^ (95% CI)	Adjusted *p*-Value
C10:0	−0.14 (−0.49, 0.20)	0.257	−0.16 (−0.43, 0.11)	0.241
C12:0	−0.79 (−2.21, 0.63)	0.219	−0.63 (−1.76, 0.49)	0.267
C14:0	−0.99 (−2.42, 0.43)	0.121	−0.45 (−1.51, 0.61)	0.399
C16:0	−2.61 (−5.58, 0.35)	0.057	−1.51 (−3.85, 0.82)	0.200
C18:0	−0.17 (−1.49, 1.14)	0.415	−0.92 (−1.95, 0.11)	0.081
C20:0	−0.02 (−0.07, 0.02)	0.564	−0.02 (−0.06, 0.01)	0.193
C22:0	0.03 (−0.01, 0.08)	0.055	0.02 (−0.01, 0.06)	0.152
C23:0	0.00 (−0.26, 0.26)	0.492	−0.01 (−0.22, 0.19)	0.902
C24:0	0.01 (−0.01, 0.03)	**0.003**	0.03 (0.01, 0.06)	**0.004**
**SFAs**	−4.42 (−9.34, 0.49)	**0.028**	−3.34 (−7.39, 0.69)	0.103
C14:1	−0.01 (−0.09, 0.07)	0.216	−0.01 (−0.07, 0.04)	0.646
C18:1n-7	−2.52 (−10.96, 5.91)	0.326	−2.17 (−7.53, 3.17)	0.417
C18:1n-9	0.29 (−9.19, 9.79)	0.575	−0.11 (−6.60, 6.37)	0.971
C20:1n-9	−0.04 (−0.14, 0.05)	0.845	−0.01 (−0.08, 0.07)	0.898
C22:1n-9	−0.01 (−0.16, 0.12)	0.512	0.08 (−0.04, 0.20)	0.182
**MUFAs**	−2.14 (−7.01, 2.71)	0.540	−2.39 (−5.76, 0.97)	0.160
C18:2n-6 (LA)	3.01 (−2.58, 8.62)	0.140	2.86 (−1.10, 6.83)	0.153
C18:3n-6 (GLA)	−0.01 (−0.05, 0.03)	0.762	−0.01 (−0.04, 0.02)	0.664
C20:2n-6	0.21 (−0.11, 0.54)	0.138	0.19 (−0.09, 0.47)	0.180
C20:3n-6	−0.01 (−0.12, 0.08)	0.773	0.01 (−0.07, 0.10)	0.719
C20:4n-6 (AA)	0.09 (−0.03, 0.22)	**0.009**	0.12 (0.02, 0.21)	**0.013**
C22:2n-6	0.09 (−1.96, 2.15)	0.683	−0.14 (−1.96, 1.66)	0.873
C22:4n-6	0.04 (−0.07, 0.16)	**0.041**	0.08 (−0.01, 0.16)	0.065
C22:5n-6	0.09 (0.02, 0.16)	**0.010**	0.06 (0.01, 0.12)	**0.015**
**N-6**	4.60 (−1.78, 10.99)	0.155	3.73 (−0.83, 8.30)	0.107
C18:3n-3 (ALA)	0.78 (0.05, 1.52)	**0.036**	0.44 (−0.17, 1.07)	0.155
C20:5n-3 (EPA)	−0.04 (−0.15, 0.06)	0.902	−0.04 (−0.12, 0.04)	0.319
C22:5n-3	−0.01 (−0.14, 0.11)	0.155	0.06 (−0.04, 0.16)	0.264
C22:6n-3 (DHA)	0.11 (−0.09, 0.31)	0.06	0.11 (−0.04, 0.27)	0.171
**N-3**	0.83 (−0.03, 1.70)	**0.014**	0.67 (−0.02, 1.37)	0.059
**PUFAs**	5.21 (−1.28, 11.71)	0.060	4.27 (−0.46, 9.01)	0.076
**N6/N3**	−5.51 (−18.27, 7.24)	0.321	−4.07 (−13.48, 5.33)	0.388

^a^ Unadjusted linear mixed model based on the terms time (i.e., stage of lactation), infant health status variable and variable * time (Infants without health condition are the reference group). ^b^ Adjusted for time, maternal age, mother’s post-pregnancy BMI, infant’s feeding pattern, infant sex.

## Data Availability

The data presented in this study are available on request from the corresponding author. The data are not publicly available due to ethical concerns related to the confidentiality of participants.

## Supplementary Materials

The following supporting information can be downloaded at: https://www.mdpi.com/article/10.3390/cells11244110/s1, Figure S1: Example of gating strategy; Figure S2: Flow diagram showing the recruitment process and study participants at baseline and follow-up; Figure S3: A comparison of CD64 marker expression levels on hmPMNs in the human milk of mothers of a single infant who was infected/diseased and a single infant who was healthy; Figure S4: Simplified Diagram comparing the activation states, based on the CD markers, of oPMNs and hmPMNs, both early and at 4-months postpartum; Figure S5: Diagram comparing the (A) activation states, based on the CD markers, of hmPMNs and the changes in PUFAs levels during lactation (from baseline until 4-months postpartum), among mothers with moderate and/or severe oral inflammatory load (OIL); Table S1: Dietary intake of lactating women across the health, moderate and severe OIL groups; Table S2: Mean ± SD Values comparing Neutrophils and CD biomarkers in human milk and oral cavity at baseline and follow-up; Table S3: Differences in the absolute hmPMN and CD markers levels in OIL groups at baseline and follow-up; Table S4: Human milk fatty acids profile according to maternal oral inflammatory load state at baseline and follow-up; Table S5: Correlation between hmPMNs and CD markers and fatty acids levels in human milk at baseline; Table S6: Correlation between hmPMNs and CD markers and fatty acids levels in human milk at follow-up.
