# Supplementary material for: The Association between Maternal Oral Inflammation and Neutrophil Phenotypes and Poly-Unsaturated Fatty Acids Composition in Human Milk: A Prospective Cohort Study
Source: Cells. 2022 Dec 17;11(24):4110. doi: 10.3390/cells11244110 (PMC9777263; doi:10.3390/cells11244110)
Supplement: Supplementary file 1 [file cells-11-04110-s001.zip › Supplementary Figure S1.pdf]

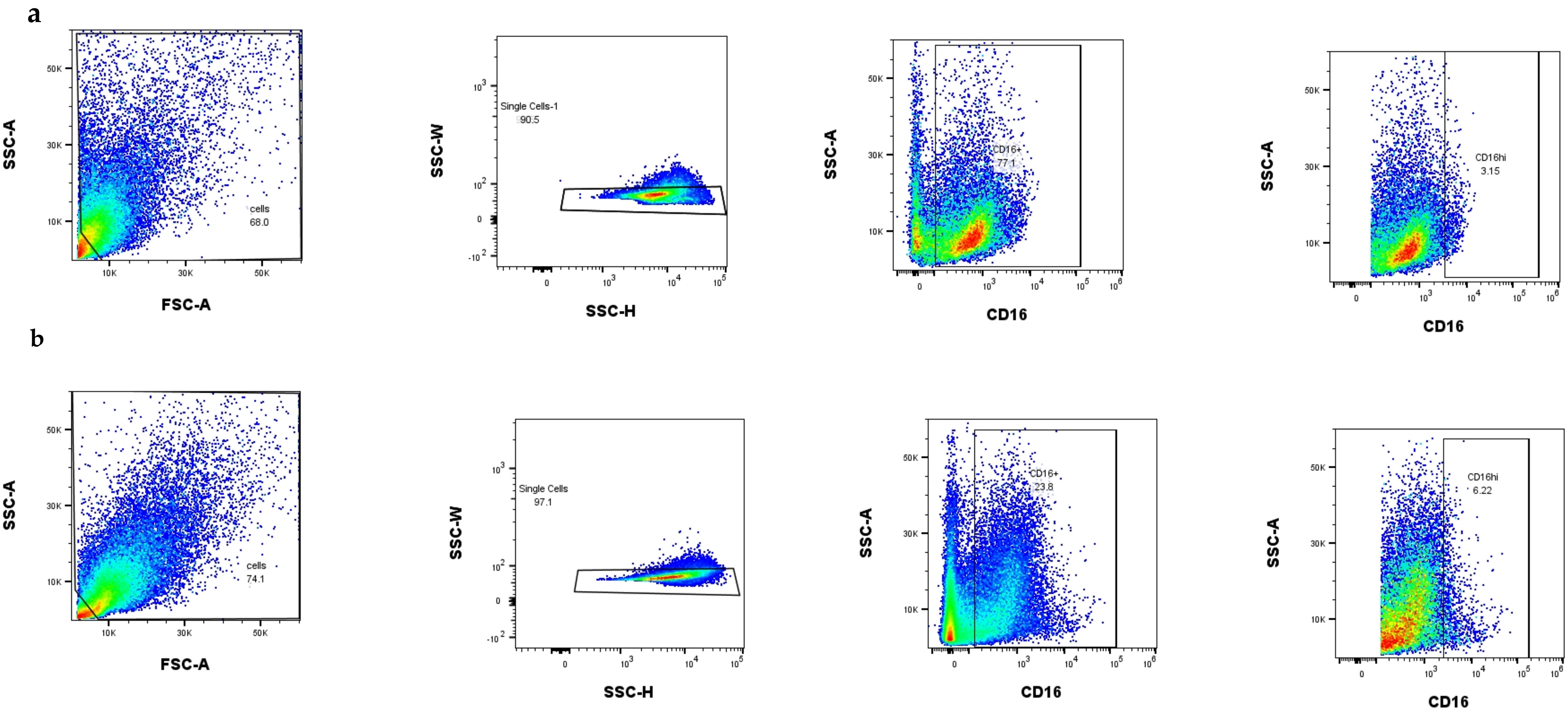

Supplementary Figure S1. Example of gating strategy used for a single participant's oral rinse sample (a) and a single participant's human milk sample (b) used in the FlowJo software to measure the geometric mean fluorescence intensity (MFI) for each of the polymorphonuclear neutrophils (PMNs) markers. Gating was performed as follows: Doublets were excluded by SSC-W × SSC-H (side scatter: width × height). To ensure that 100% of the PMNs are captured, a CD16+ area was utilized (CD16+/SSC-A). Oral and human milk PMNs were then gated based on high CD16 expression in combination with side scatter area ($CD16^{hi}$ /SSC-A), as shown in the last gating scheme.
