# Supplementary material for: The Association between Maternal Oral Inflammation and Neutrophil Phenotypes and Poly-Unsaturated Fatty Acids Composition in Human Milk: A Prospective Cohort Study
Source: Cells. 2022 Dec 17;11(24):4110. doi: 10.3390/cells11244110 (PMC9777263; doi:10.3390/cells11244110)
Supplement: Supplementary file 1 [file cells-11-04110-s001.zip › Supplementary Figure S3.pdf]

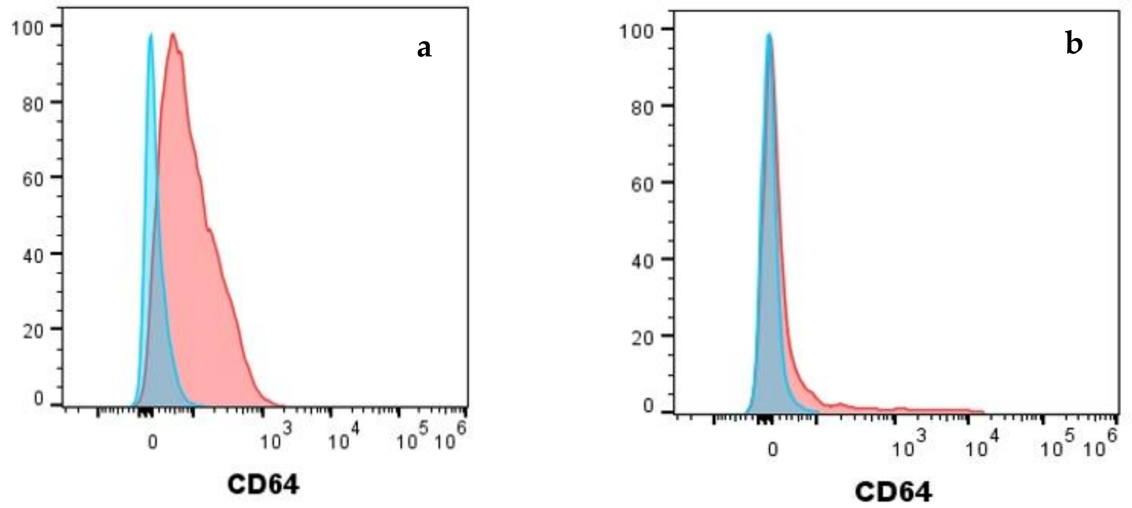

Supplementary Figure S3. A comparison of CD64 marker expression levels on hmPMNs in the human milk of mothers of a single infant who was infected/diseased (n=1) (a) and a single infant who was healthy (n=1) (b). Representative histograms showing the expression levels of CD64 biomarker on hmPMNs of a mother of a single infected infant (a) and a single healthy infant (b). (a) Higher expression levels of CD64 marker were observed when the infant was infected (histogram in red) and the levels return back to normal upon recovery (histogram in blue), but (b) this change was not observed when the infant was healthy during lactation .
