# Supplementary material for: The Association between Maternal Oral Inflammation and Neutrophil Phenotypes and Poly-Unsaturated Fatty Acids Composition in Human Milk: A Prospective Cohort Study
Source: Cells. 2022 Dec 17;11(24):4110. doi: 10.3390/cells11244110 (PMC9777263; doi:10.3390/cells11244110)
Supplement: Supplementary file 1 [file cells-11-04110-s001.zip › Supplementary Figure S4.pdf]

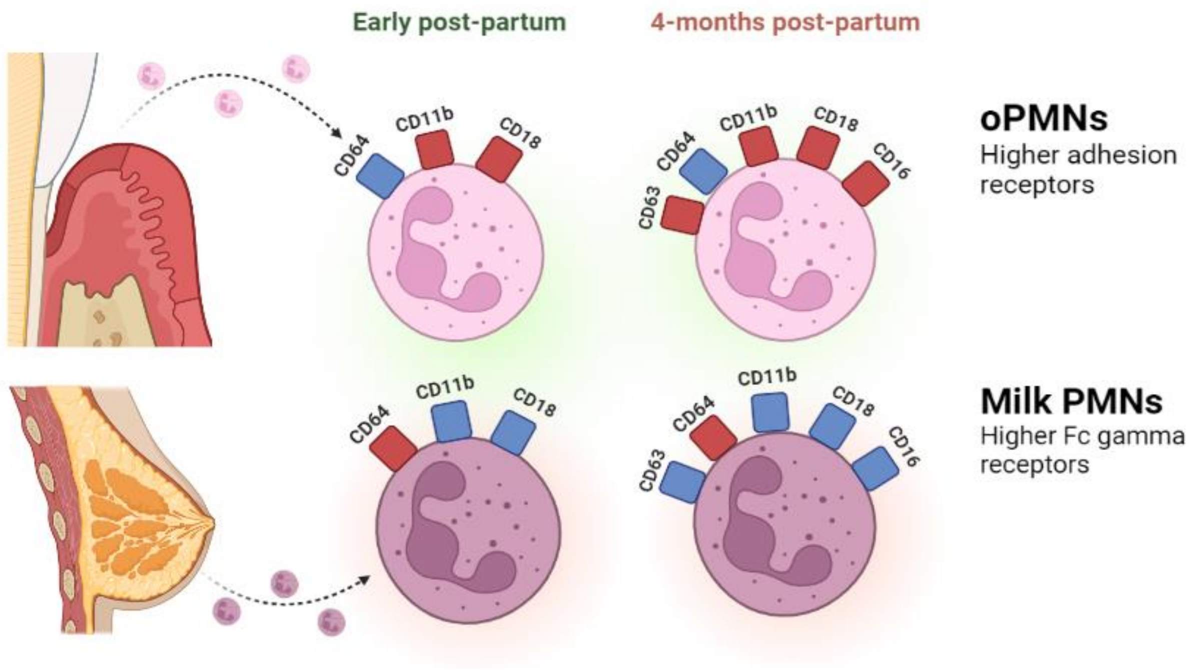

Supplementary Figure S4. Simplified Diagram comparing the activation states, based on the CD markers, of oPMNs and hmPMNs, both early and at 4-months *postpartum*. Several markers responsible for adhesion (namely: CD11b, CD16 and CD18), and markers of activation/degranulation (namely: CD63) are highly expressed on oPMNs (marked in red) compared to hmPMNs (marked in blue). However, the Fc gamma-receptor I (namely: CD64) is down-regulated on oPMNs (marked in blue) in comparison to the hmPMNs (marked in red). The data are based on flow cytometry results.
