# Supplementary material for: The Association between Maternal Oral Inflammation and Neutrophil Phenotypes and Poly-Unsaturated Fatty Acids Composition in Human Milk: A Prospective Cohort Study
Source: Cells. 2022 Dec 17;11(24):4110. doi: 10.3390/cells11244110 (PMC9777263; doi:10.3390/cells11244110)
Supplement: Supplementary file 1 [file cells-11-04110-s001.zip › Supplementary Figure S5.pdf]

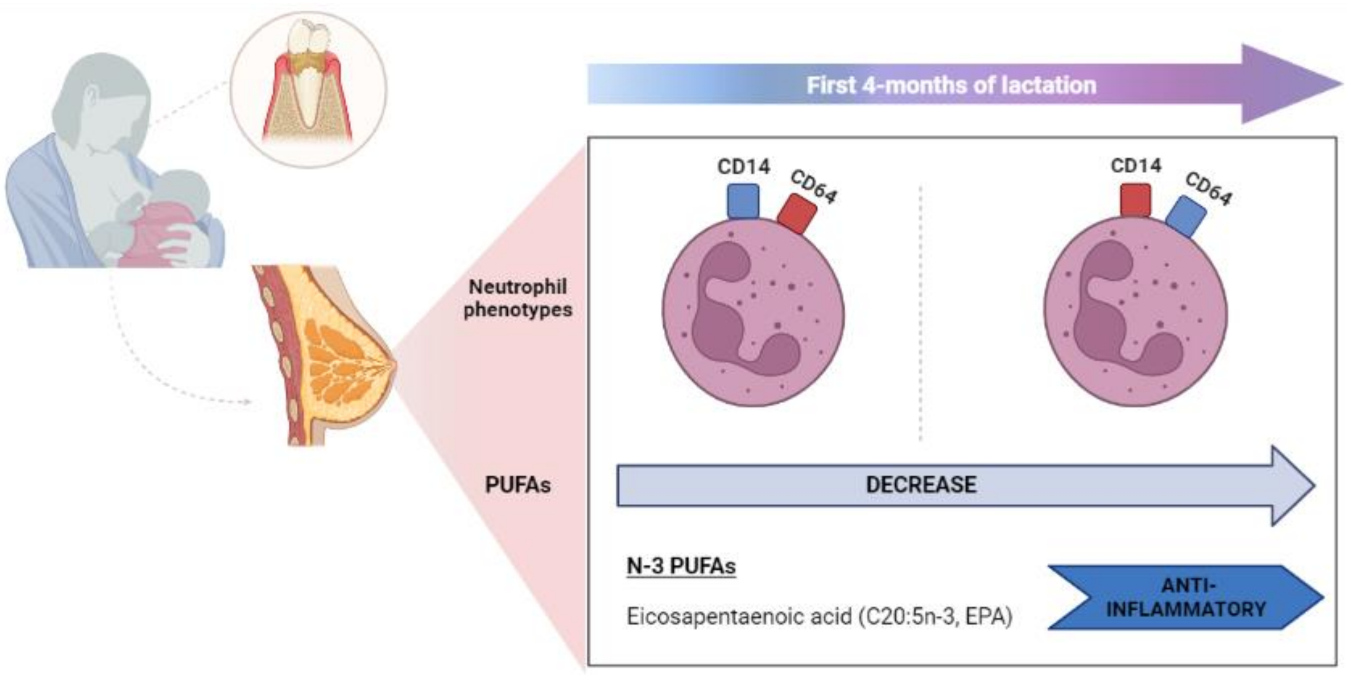

Supplementary Figure S5. Diagram comparing the (A) activation states, based on the CD markers, of hmPMNs and the (B) changes in PUFAs levels during lactation (from baseline until 4-months postpartum), among mothers with moderate to severe oral inflammatory load (OIL). (A) The marker responsible for lipopolysaccharide (LPS) binding (namely: CD14) was upregulated on hmPMNs at 4-months postpartum (marked in red) compared to early postpartum (marked in blue), whereas the marker of activation/degranulation (namely: CD64) was down-regulated on hmPMNs at 4-months postpartum (marked in blue) compared to early postpartum (marked in red). These changes were significantly different from the expression levels of CD14 and CD64 on hmPMNs of mothers with healthy OIL (B) There was a significant decrease in the levels of anti-inflammatory (marked in blue) n-3 PUFA (EPA) at follow-up compared to baseline. These changes were also statistically significantly different from the levels of EPA in human milk of mothers with healthy OIL.
