# Supplementary material for: The Association between Maternal Oral Inflammation and Neutrophil Phenotypes and Poly-Unsaturated Fatty Acids Composition in Human Milk: A Prospective Cohort Study
Source: Cells. 2022 Dec 17;11(24):4110. doi: 10.3390/cells11244110 (PMC9777263; doi:10.3390/cells11244110)
Supplement: Supplementary file 1 [file cells-11-04110-s001.zip › Supplementary Table S1.pdf]

Supplementary Table S1. Dietary intake of lactating women across the health, moderate and severe OIL groups

Maternal dietary intake	Baseline (n=40)				Follow-up (n=42)			
	Healthy (n=17)	Moderate (n=6)	Severe (n=17)	p-value	Healthy (n=13)	Moderate (n=12)	Severe (n=17)	p-value
	Mean (SD)	Mean (SD)	Mean (SD)		Mean (SD)	Mean (SD)	Mean (SD)	
**Energy (kcal/day)**	2129.97 (761.19)	2537.97 (618.55)	2032.67 (876.87)	0.415[a]	2020.27 (877.75)	2196.24 (524.10)	1816.29 (585.74)	0.250
**Total lipids (g/day)**	87.51 (45.76)	106.93 (41.24)	83.24 (63.80)	0.361	79.86 (47.75)	94.72 (35.54)	72.22 (34.83)	0.321
**SFAs (g)**	33.93 (20.97)	32.08 (15.53)	25.90 (17.56)	0.415	25.43 (15.46)	33.45 (19.73)	23.57 (13.00)	0.347
**MUFAs (g)**	29.50 (17.24)	41.55 (17.35)	31.35 (26.17)	0.204	29.29 (21.88)	34.13 (13.79)	28.06 (14.82)	0.393
**PUFAs (g)**	15.77 (7.64)	23.74 (9.51)	18.78 (20.43)	0.155	17.72 (12.02)	19.17 (9.73)	14.92 (7.16)	0.618
**Eicosapentaenoic acid (EPA) (g)**	0.03 (0.05)	0.38 (0.51)	0.12 (0.35)	0.171	0.11 (0.23)	0.11 (0.31)	0.004 (0.005)	**0.007***
**Docosapentaenoic acid (DPA) (g)**	0.02 (0.01)	0.11 (0.15)	0.04 (0.08)	0.113	0.04 (0.08)	0.03 (0.07)	0.01 (0.01)	0.168
**Docosahexaenoic acid (DHA) (g)**	0.05 (0.07)	0.59 (0.75)	0.21 (0.55)	0.068	0.24 (0.56)	0.22 (0.59)	0.02 (0.03)	0.123

[a] One-way ANOVA. * Differences between Severe and Healthy, and Severe and Moderate OIL groups.
