# Supplementary material for: The Association between Maternal Oral Inflammation and Neutrophil Phenotypes and Poly-Unsaturated Fatty Acids Composition in Human Milk: A Prospective Cohort Study
Source: Cells. 2022 Dec 17;11(24):4110. doi: 10.3390/cells11244110 (PMC9777263; doi:10.3390/cells11244110)
Supplement: Supplementary file 1 [file cells-11-04110-s001.zip › Supplementary Table S2.pdf]

Supplementary Table S2. Mean ± SD Values comparing Neutrophils and CD biomarkers in human milk and oral cavity at baseline and follow-up

Neutrophils and CD biomarkers[†]	Baseline (N=48*)		P-value	Follow-up (N=46)		P-value
	Oral Cavity	Human Milk		Oral Cavity	Human Milk	
**Absolute PMN count ($x10^6$/10ml)**	1.29 ± 1.59	0.11 ± 0.40	**0.000**[a]	1.93 ± 4.24	0.14 ± 0.4	**0.000**[a]
**CD16**	474.5 ± 190.5	512.7 ± 354.5	0.406	507.0 ± 182.3	399.7 ± 193.4	**0.001**[a]
**CD66a**	479.1 ± 162.6	468.9 ± 201.3	0.457	469.8 ± 153.3	426.5 ± 146.9	0.112
**CD11b**	601.8 ± 278.9	493.6 ± 225.1	**0.020**[a]	548.4 ± 250.6	376.4 ± 167.9	**0.000**[a]
**CD18**	5537.6 ± 2409.5	4122.0 ± 1879.8	**0.000**[a]	4108.4 ± 3148.2	2823.8 ± 1431.7	**0.001**[a]
**CD64**	41.9 ± 17.2	92.0 ± 66.2	**0.000**[a]	45.9 ± 41.2	72.1 ± 106.4	**0.016**[a]
**CD63**	300.9 ± 124.1	311.3 ± 168.1	0.825	270.1 ± 111.1	236.7 ± 100.9	**0.042**[a]

[a] Significance level= 0.05. Data are presented as Mean ± SD
*N=48 instead of 50. Since two of the human milk samples were excluded at baseline due to the unavailability of data from the flow cytometry
[†] For the CD markers, data is presented as the geometric mean fluorescence intensity (MFI) for each CD marker
