# Supplementary material for: The Association between Maternal Oral Inflammation and Neutrophil Phenotypes and Poly-Unsaturated Fatty Acids Composition in Human Milk: A Prospective Cohort Study
Source: Cells. 2022 Dec 17;11(24):4110. doi: 10.3390/cells11244110 (PMC9777263; doi:10.3390/cells11244110)
Supplement: Supplementary file 1 [file cells-11-04110-s001.zip › Supplementary Table S3.pdf]

Supplementary Table S3. Differences in the absolute hmPMN and CD markers levels in OIL groups at baseline and follow-up

Neutrophils and CD biomarkers†	Baseline (N=48[a])				Follow-up (N=46)			
	Healthy (n=20)	Moderate Inflammation (n=7)	Severe Inflammation (n=21)	P-value	Healthy (n=16)	Moderate Inflammation (n=13)	Severe Inflammation (n=17)	P-value
**Absolute hmPMN count ($x10^6$/10ml)**	0.17 ± 0.58	0.19 ± 0.19	0.07 ± 0.25	0.850	0.05 ± 0.07	0.22 ± 0.64	0.16 ± 0.27	0.407
**CD16**	469.1 ± 184.9	529.1 ± 223.2	548.7 ± 494.5	0.814	483.9 ± 263.8	326.5 ± 82.5	376.4 ± 151.8	0.148
**CD66a**	411.6 ± 157.0	541.1 ± 284.3	499.5 ± 203.5	0.278	438.3 ± 168.7	437.4 ± 116.3	407.2 ± 152.4	0.667*
**CD11b**	522.3 ± 307.6	513.9 ± 120.0	459.6 ± 150.7	0.510	428.2 ± 207.3	364.5 ± 126.2	336.7 ± 150.2	0.334
**CD18**	4193.3 ± 2108.0	3849.0 ± 1448.9	4145.0 ± 1849.7	0.915*	2569.7 ± 1115.7	3088.0 ± 1358.4	2861.0 ± 1758.3	0.666
**CD14**	174.9 ± 221.6	150.1 ± 111.8	115.3 ± 120.7	0.363	133.2 ± 106.5	189.6 ± 154.3	214.2 ± 144.6	0.274
**CD64**	72.8 ± 28.6	122.9 ± 87.7	100.6 ± 80.3	0.556	94.6 ± 168.8	65.2 ± 70.6	56.1 ± 28.7	0.606
**CD63**	284.3 ± 144.1	331.9 ± 198.6	330.1 ± 183.3	0.789	241.3 ± 116.7	269.2 ± 92.7	207.4 ± 88.3	0.289*

*One-way ANOVA (for normally-distributed data)

† For the CD markers, data is presented as the geometric mean fluorescence intensity (MFI) for each CD marker

[a] N=48 instead of 50. Since two of the human milk samples were excluded at baseline due to the unavailability of data from the flow cytometry

Data are presented as Mean ± SD
