# Supplementary material for: The Association between Maternal Oral Inflammation and Neutrophil Phenotypes and Poly-Unsaturated Fatty Acids Composition in Human Milk: A Prospective Cohort Study
Source: Cells. 2022 Dec 17;11(24):4110. doi: 10.3390/cells11244110 (PMC9777263; doi:10.3390/cells11244110)
Supplement: Supplementary file 1 [file cells-11-04110-s001.zip › Supplementary Table S4.pdf]

Supplementary Table S4. Human milk fatty acids profile according to maternal oral inflammatory load state at baseline and follow-up

**Fatty acids in human milk**	**Baseline (n=50)**					**Follow-up (n=46)**				
	**Healthy (n=21)**	**Moderate (n=8)**	**Severe (n=21)**	**Unadjusted *p*-value**	**Adjusted *p*-value[b]**	**Healthy (n=16)**	**Moderate (n=13)**	**Severe (n=17)**	**Unadjusted *p*-value**	**Adjusted *p*-value[b]**
	**Mean (SD)**	**Mean (SD)**	**Mean (SD)**			**Mean (SD)**	**Mean (SD)**	**Mean (SD)**		
C10:0	0.76 (0.53)	0.90 (0.39)	0.88 (0.44)	0.393	0.247	0.68 (0.35)	0.60 (0.42)	0.67 (0.34)	0.793	0.479
C12:0	5.06 (1.64)	5.82 (2.55)	5.49 (2.00)	0.510	0.427	4.96 (1.01)	5.21 (2.25)	4.44 (2.13)	0.243	0.668
C14:0	5.78 (1.60)	5.92 (2.30)	5.90 (1.88)	0.949	0.895	5.85 (1.73)	6.30 (2.45)	4.85 (2.18)	0.122	0.280
C16:0	19.25 (3.90)	17.76 (2.51)	18.39 (2.81)	0.476	0.113	18.91 (3.92)	18.41 (3.98)	18.08 (3.52)	0.825	0.339
C18:0	7.22 (1.37)	6.61 (1.29)	6.74 (1.09)	0.155	0.330	6.86 (1.67)	7.39 (2.19)	7.33 (1.41)	0.652	0.879
C20:0	0.15 (0.06)	0.14 (0.03)	0.15 (0.06)	0.865	0.831	0.13 (0.04)	0.15 (0.07)	0.16 (0.05)	0.479	0.231
C22:0	0.06 (0.06)	0.06 (0.05)	0.06 (0.08)	0.598	0.877	0.02 (0.03)	0.02 (0.02)	0.03 (0.04)	0.152	0.051
C23:0	0.24 (0.42)	0.42 (0.37)	0.25 (0.24)	0.163	0.684	0.33 (0.33)	0.24 (0.25)	0.16 (0.24)	0.114	0.068
C24:0	0.04 (0.05)	0.05 (0.04)	0.04 (0.05)	0.631	0.762	0.01 (0.01)	0.01 (0.02)	0.02 (0.06)	0.300	0.135
**SFAs**	38.54 (6.55)	37.72 (5.16)	37.94 (6.02)	0.706	0.440	37.77 (5.75)	38.37 (4.28)	35.78 (7.65)	0.481	0.296
C14:1	0.25 (0.11)	0.21 (0.11)	0.25 (0.12)	0.768	0.773	0.26 (0.14)	0.18 (0.09)	0.20 (0.11)	0.372	0.274
C18:1n-7	5.94 (11.78)	6.77 (13.18)	2.18 (0.32)	0.890	0.607	7.52 (15.31)	1.93 (0.63)	2.15 (0.23)	0.612	0.196
C18:1n-9	34.73 (12.58)	32.02 (13.38)	39.53 (4.83)	0.199	0.362	35.91 (15.19)	42.16 (6.89)	43.81 (5.00)	0.152	0.051
C20:1n-9	0.46 (0.15)	0.43 (0.08)	0.43 (0.12)	0.721	0.363	0.39 (0.19)	0.38 (0.08)	0.40 (0.11)	0.610	0.980
C22:1n-9	0.09 (0.11)	0.12 (0.07)	0.07 (0.06)	0.181	0.461	0.14 (0.34)	0.07 (0.08)	0.03 (0.05)	0.160	0.223
**MUFAs**	41.47 (5.14)	39.56 (3.39)	42.47 (5.06)	0.889	0.393	44.24 (6.56)	44.74 (6.73)	46.61 (5.08)	0.509	0.186
C18:2n-6 (LA)	12.79 (5.69)	15.13 (2.58)	13.16 (6.84)	0.762	0.981	11.86 (6.16)	10.79 (7.52)	12.17 (8.21)	0.303	0.946
C18:3n-6 (GLA)	0.15 (0.06)	0.15 (0.06)	0.12 (0.06)	0.354	0.533	0.12 (0.04)	0.11 (0.06)	0.12 (0.04)	0.456	0.631

C20:2n-6	0.40 (0.73)	0.35 (0.16)	0.33 (0.21)	0.286	0.768	0.21 (0.12)	0.24 (0.14)	0.19 (0.11)	0.685	0.663
C20:3n-6	0.63 (0.18)	0.58 (0.13)	0.55 (0.14)	0.151	0.236	0.35 (0.05)	0.36 (0.11)	0.35 (0.07)	0.960	0.684
C20:4n-6 (AA)	0.72 (0.19)	0.77 (0.19)	0.73 (0.18)	0.608	0.649	0.57 (0.15)	0.64 (0.14)	0.62 (0.09)	0.359	0.750
C22:2n-6	2.81 (4.59)	2.49 (1.45)	2.05 (1.28)	0.350	0.666	2.41 (1.47)	2.24 (1.21)	1.71 (0.99)	0.315	0.113
C22:4n-6	0.11 (0.14)	0.29 (0.21)	0.17 (0.15)	0.070	**0.018***	0.14 (0.16)	0.12 (0.11)	0.12 (0.14)	0.902	0.459
C22:5n-6	0.08 (0.06)	0.15 (0.06)	0.13 (0.09)	**0.036**[a]	**0.026***	0.08 (0.06)	0.12 (0.12)	0.05 (0.04)	0.217	0.909
**N-6**	17.69 (7.50)	19.94 (3.52)	17.27 (7.37)	0.813	0.820	15.77 (6.91)	14.65 (8.47)	15.36 (8.91)	0.794	0.847
C18:3n-3 (ALA)	1.67 (0.68)	1.86 (0.43)	1.54 (0.88)	0.390	0.344	1.64 (0.89)	1.80 (0.86)	1.79 (1.09)	0.615	0.390
C20:5n-3 (EPA)	0.07 (0.12)	0.17 (0.16)	0.11 (0.14)	0.228	0.291	0.14 (0.15)	0.07 (0.07)	0.06 (0.09)	0.408	0.286
C22:5n-3	0.11 (0.17)	0.23 (0.22)	0.15 (0.15)	0.289	0.490	0.13 (0.14)	0.12 (0.13)	0.06 (0.13)	0.443	0.120
C22:6n-3 (DHA)	0.43 (0.30)	0.49 (0.31)	0.48 (0.30)	0.547	0.362	0.26 (0.11)	0.23 (0.19)	0.29 (0.19)	0.509	0.942
**N-3**	2.29 (0.83)	2.76 (1.00)	2.30 (1.12)	0.619	0.209	2.19 (1.01)	2.23 (0.95)	2.22 (1.17)	0.773	0.682
**PUFAs**	19.98 (7.46)	22.71 (3.67)	19.58 (7.83)	0.587	0.984	17.97 (7.64)	16.88 (8.78)	17.59 (8.98)	0.843	0.895
**N6/N3**	10.05 (8.44)	7.81 (2.45)	10.48 (13.37)	0.986	0.858	8.45 (5.46)	11.90 (21.98)	13.46 (20.79)	0.680	0.375
