# Supplementary material for: The Association between Maternal Oral Inflammation and Neutrophil Phenotypes and Poly-Unsaturated Fatty Acids Composition in Human Milk: A Prospective Cohort Study
Source: Cells. 2022 Dec 17;11(24):4110. doi: 10.3390/cells11244110 (PMC9777263; doi:10.3390/cells11244110)
Supplement: Supplementary file 1 [file cells-11-04110-s001.zip › Supplementary Table S5.pdf]

Supplementary Table S5. Correlation between hmPMNs and CD markers and the fatty acids levels in human milk at baseline

**Fatty acids in human milk**	**Human milk neutrophils (x10^6/10ml)**	**CD16**	**CD66a**	**CD11b**	**CD18**	**CD14**	**CD64**	**CD63**
C10:0	0.135	0.038	**0.453***	**0.326***	0.194	0.216	0.051	**0.447****
C12:0	0.268	0.057	**0.362***	0.074	**0.411****	-0.056	0.017	**0.307***
C14:0	0.078	-0.080	0.091	0.016	0.277	0.013	-0.074	0.111
C16:0	-0.053	-0.136	0.037	0.128	0.044	0.142	**-0.326***	0.037
C18:0	0.110	0.008	0.009	0.063	-0.153	0.116	-0.113	0.073
C20:0	-0.007	0.029	0.277	0.258	0.013	0.195	-0.057	0.146
C22:0	0.024	-0.128	-0.024	-0.068	0.033	-0.209	-0.246	-0.067
C23:0	0.001	0.151	0.051	-0.029	-0.045	**-0.329***	-0.052	-0.057
C24:0	0.146	-0.243	-0.049	-0.180	0.156	**-0.327***	-0.191	-0.069
**SFAs**	0.049	-0.098	0.146	0.088	0.182	0.108	-0.116	0.141
C14:1	-0.086	-0.216	-0.104	0.036	-0.020	-0.060	**-0.383****	-0.006
C18:1n-7	-0.138	-0.062	-0.001	0.148	-0.229	0.172	-0.133	-0.098
C18:1n-9	-0.101	-0.060	-0.147	-0.170	-0.264	-0.068	0.059	**-0.288***
C20:1n-9	0.208	0.258	-0.026	0.116	0.007	0.166	0.082	-0.156
C22:1n-9	0.115	-0.005	0.054	0.036	**0.318***	-0.192	-0.090	-0.082
**MUFAs**	-0.063	0.004	-0.103	-0.031	-0.270	0.069	0.022	-0.252
C18:2n-6 (LA)	0.030	0.170	-0.165	-0.062	-0.021	-0.013	0.219	-0.110
C18:3n-6 (GLA)	0.136	-0.022	-0.064	-0.006	0.127	0.179	0.071	-0.133
C20:2n-6	0.079	-0.099	0.094	-0.125	**0.291***	0.018	0.158	0.088
C20:3n-6	0.119	-0.044	-0.150	-0.178	0.105	0.125	-0.089	-0.150
C20:4n-6 (AA)	0.056	-0.046	-0.024	-0.043	0.000	0.126	-0.097	-0.022
C22:2n-6	0.054	0.139	-0.140	-0.084	-0.012	**-0.525*****	-0.071	-0.106
C22:4n-6	-0.037	0.006	0.129	-0.153	0.109	**-0.484*****	-0.111	-0.142

C22:5n-6	-0.047	-0.131	-0.089	-0.135	-0.163	0.075	0.120	-0.023
**N-6**	0.001	0.157	-0.151	-0.121	0.049	-0.137	0.134	-0.074
C18:3n-3 (ALA)	-0.033	0.157	-0.073	0.082	-0.088	-0.035	0.077	-0.142
C20:5n-3 (EPA)	-0.150	0.048	0.046	-0.069	0.095	**-0.345***	-0.077	-0.062
C22:5n-3	-0.095	0.029	0.012	-0.248	0.066	**-0.455****	-0.086	-0.178
C22:6n-3 (DHA)	0.190	-0.085	0.222	0.099	0.282	0.120	0.068	0.147
**N-3**	0.004	0.194	0.049	0.057	0.027	-0.139	0.060	-0.059
**PUFAs**	-0.047	0.117	-0.072	-0.101	0.038	-0.145	0.151	-0.045
**N6/N3**	-0.004	-0.001	-0.194	-0.096	-0.030	-0.066	0.017	-0.005

Data presented as spearman's correlation coefficients between hmPMNs and CD biomarkers and FAs composition in breast milk. * represent p-values ≤0.05, ** represent p-values <0.01, ***represent p-values <0.001.
