# Supplementary material for: The Association between Maternal Oral Inflammation and Neutrophil Phenotypes and Poly-Unsaturated Fatty Acids Composition in Human Milk: A Prospective Cohort Study
Source: Cells. 2022 Dec 17;11(24):4110. doi: 10.3390/cells11244110 (PMC9777263; doi:10.3390/cells11244110)
Supplement: Supplementary file 1 [file cells-11-04110-s001.zip › Supplementary Table S6.pdf]

Supplementary Table S6. Correlation between hmPMNs and CD markers and the fatty acids levels in human milk at follow-up

Fatty acids in human milk	Human milk neutrophils ($x10^6$/10ml)	CD16	CD66a	CD11b	CD18	CD14	CD64	CD63
C10:0	0.084	-0.056	0.056	-0.070	-0.120	-0.013	0.159	0.017
C12:0	0.034	0.023	-0.076	-0.012	-0.152	-0.001	-0.147	0.008
C14:0	-0.170	-0.045	0.026	-0.054	-0.017	0.036	-0.120	0.084
C16:0	0.073	**0.292***	0.140	0.093	-0.223	0.216	-0.056	-0.166
C18:0	0.090	0.170	-0.059	-0.009	-0.138	**0.311***	-0.058	**-0.396****
C20:0	**0.397****	0.148	-0.124	-0.063	-0.108	0.153	0.081	**-0.331***
C22:0	-0.221	-0.242	0.036	-0.062	**-0.514****	-0.079	-0.049	-0.001
C23:0	**-0.335***	**-0.370***	-0.064	-0.158	**-0.353***	**-0.558****	0.158	0.131
C24:0	-0.271	**-0.348***	-0.032	0.027	0.120	-0.048	0.284	0.244
**SFAs**	-0.018	0.239	0.101	0.067	-0.244	**0.304***	-0.051	-0.097
C14:1	**-0.296***	-0.064	0.043	-0.186	-0.090	0.009	-0.036	-0.042
C18:1n-7	**0.393****	0.245	0.218	0.132	0.197	-0.065	-0.165	-0.017
C18:1n-9	**0.501****	0.104	-0.166	0.005	-0.064	-0.068	**0.314***	-0.242
C20:1n-9	**0.425****	0.024	0.004	0.058	0.048	-0.081	-0.035	-0.030
C22:1n-9	-0.269	-0.238	-0.109	-0.125	0.140	**-0.353***	0.073	0.163
**MUFAs**	**0.586*****	0.232	-0.032	0.131	-0.085	-0.007	**0.323***	-0.181
C18:2n-6 (LA)	**-0.321***	**-0.337***	-0.085	-0.203	0.052	-0.004	0.279	0.142
C18:3n-6 (GLA)	-0.053	0.021	-0.028	-0.226	0.085	-0.171	-0.216	0.061
C20:2n-6	**-0.338***	-0.239	-0.036	-0.001	**0.344***	-0.274	0.065	0.233
C20:3n-6	0.015	0.000	-0.176	-0.231	-0.075	-0.153	**0.335***	0.028
C20:4n-6 (AA)	0.008	0.273	0.045	0.149	0.120	0.038	-0.114	-0.050
C22:2n-6	**-0.484****	**-0.350***	-0.067	-0.123	0.269	-0.287	0.197	0.122
C22:4n-6	-0.182	-0.217	0.184	0.005	0.226	-0.165	**0.312***	0.278
C22:5n-6	-0.190	-0.121	0.024	-0.015	**0.321***	-0.231	-0.134	0.140

**N-6**	**-0.381****	**-0.351***	-0.041	-0.171	0.160	-0.101	0.282	0.210
C18:3n-3 (ALA)	-0.239	-0.202	0.020	0.075	0.251	-0.180	-0.042	0.221
C20:5n-3 (EPA)	**-0.295***	**-0.332***	-0.158	-0.178	**0.367***	**-0.501****	0.107	0.020
C22:5n-3	**-0.398****	**-0.379****	-0.108	-0.067	0.287	**-0.467****	0.132	0.197
C22:6n-3 (DHA)	0.109	0.265	-0.117	0.211	0.063	0.130	0.021	-0.114
**N-3**	-0.242	-0.210	-0.041	0.089	**0.315***	-0.284	-0.008	0.226
**PUFAs**	**-0.440****	**-0.393****	-0.054	-0.179	0.241	-0.169	0.236	0.213
**N6/N3**	-0.117	-0.044	0.004	-0.213	-0.127	0.066	0.124	0.073

Data presented as spearman's correlation coefficients between hmPMNs and CD biomarkers and FAs composition in breast milk. * represent p-values ≤0.05, ** represent p-values <0.01, ***represent p-values <0.001
